# Supplementary material for: Early detection of emerging viral variants through analysis of community structure of coordinated substitution networks
Source: Nat Commun. 2024 Apr 2;15:2838. doi: 10.1038/s41467-024-47304-6 (PMC10987511; doi:10.1038/s41467-024-47304-6)
Supplement: Supplementary file 1 — Supplementary Information [file 41467_2024_47304_MOESM1_ESM.pdf]

1 Supplement

1.1 Supplementary tables

	Complete	1st truncated	2nd truncated
VOCs/VOIs			
Non-negative lag & significant ($p < 0.05$, Student's t-test) medium-to strong negative correlation for VOCs/VOIs across all countries	70%	74%	77%
CI ρ	$[-0.97, 0.39]$	$[-0.97, -0.36]$	$[-0.97, -0.44]$
Mean ρ	-0.73	-0.74	-0.74
CI l^*, days	$[0, 168]$	$[0, 168]$	$[0, 140]$
Mean l^*, days	23.5	20.5	20.6
VOCs only			
Non-negative lag & significant ($p < 0.05$, Student's t-test) medium-to strong negative correlation for VOCs across all countries	76%	84%	89%
CI ρ	$[-0.91, -0.36]$	$[-0.95, -0.36]$	$[-0.97, -0.37]$
Mean ρ	-0.74	-0.72	-0.72
CI l^*, days	$[0, 140]$	$[0, 168]$	$[0, 168]$
Mean l^*, days	31.3	30.9	30.5

Supplementary table 1: Cross-correlation analysis for density-based p-values and prevalences of VOCs/VOIs

	Complete	1st truncated	2nd truncated
Significantly dense VOCs/VOIs (VOCs only) across all countries	61% (90%)	64% (93%)	63% (90%)
Median frequency at calling	$6 \cdot 10^{-4}$	$4 \cdot 10^{-4}$	$4 \cdot 10^{-4}$
Median prevalence at calling	$1 \cdot 10^{-3}$	$8 \cdot 10^{-4}$	$1 \cdot 10^{-3}$
$FD^{\text{prev}} > 0$ among variants called as significantly dense	47%	57%	56%
$FD^{\text{des}} > 0$ among variants called as significantly dense	47%	52%	49%
Median FD^{prev} for early calls, days	68	60	60
Median FD^{des} for early calls, days	66	48	35
Linear correlation and p-value (two-sided Student's t-test) for numbers of sequences and significantly dense VOCs/VOIs per country	0.56 (0.024)	0.59 (0.017)	0.63 (0.009)

Supplementary table 2: VOC/VOI calling as significantly dense subgraphs.

	Complete	1st truncated	2nd truncated
VOCs/VOIs identified in at least one country	5/10	5/10	5/10
Number of countries where VOCs (VOIs) were detected	1-15 (0)	1-16 (0)	1-16 (0)
Aggregated recall for VOCs/VOIs (VOCs only).	19% (38%)	22% (44%)	21% (41%)
Precision: percentage of densest communities with at least 80% similarity with VOCs/VOIs, aggregated across all countries.	31%	21%	16%
Percentage of earliest VOCs/VOIs detections with $FD^{\text{prev}} \geq 0$	40%	43%	30%
Percentage of earliest VOCs/VOIs detections with $FD^{\text{des}} \geq 0$	67%	40%	33%
Median cumulative frequency at first detection	$1.4 \cdot 10^{-3}$	$9.96 \cdot 10^{-4}$	$1.5 \cdot 10^{-3}$
Median prevalence at first detection	$1.59 \cdot 10^{-2}$	$1.75 \cdot 10^{-2}$	$2.45 \cdot 10^{-2}$
VOCs/VOIs (VOCs) with $FD^{\text{prev}} \geq 0$ in at least one country	5/10 (5/5)	5/10 (5/5)	5/10 (5/5)
VOCs/VOIs (VOCs) with $FD^{\text{des}} \geq 0$ in at least one country	5/10 (5/5)	5/10 (5/5)	5/10 (5/5)
Median FD^{prev} for early calls, days	218	30	30
Median FD^{des} for early calls, days	123	44	36

Supplementary table 3: Analysis of densest subnetworks

1.2 Analysis of densest subnetworks of coordinated substitution networks

The results are summarized in Supplementary table 3 and Supplementary Figs. 34-41. The main findings here can be summarized as follows:

- Percentages of densest communities that were at least 80% identical to the known variants ranged from 16% in the second truncated dataset to 31% in the complete dataset. All detected variants were VOCs.

- Among these communities, 33% − 67% were early detected before the corresponding VOCs received official designation from WHO. Similarly, 30% − 40% were early detected before the VOCs attained a 1% prevalence.
- For the three datasets, the median cumulative frequencies of VOCs at times of their early detection ranged from $5 \cdot 10^{-4}$ to $9 \cdot 10^{-4}$. Respective median prevalences were between $3 \cdot 10^{-3}$ and $9 \cdot 10^{-3}$.
- Across all datasets, every VOC was detected with a minimum 0.8 accuracy in at least one country before its official designation.
- Maximal forecasting depths FD^{des} relative to the WHO designation were $231, 111, 150, 270, 319$ days (complete dataset), $231, 111, 135, 285, 319$ days (first truncated dataset), $36, 111, 45, 285, 4$ days (second truncated dataset). Median depths varied between $FD^{\text{des}} = 123$ for the complete dataset and $FD^{\text{des}} = 44$ and $FD^{\text{des}} = 36$ for truncated datasets.
- The numbers for early detection in relation to the 1% prevalence were similar, except for the Beta variant. This was detected as soon as it reached the 1% mark ($FD^{\text{prev}} = 0$).

1.3 Supplementary figures

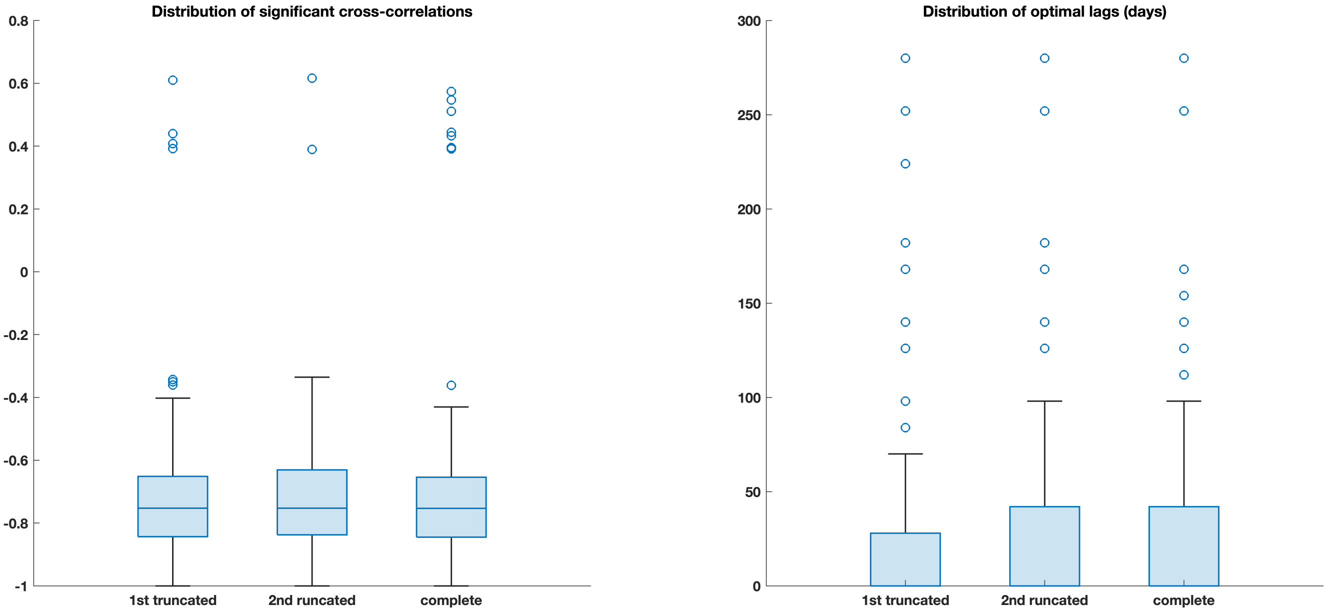

Supplementary Fig. 1: Distributions of statistically significant cross-correlations and optimal lags for three analyzed datasets

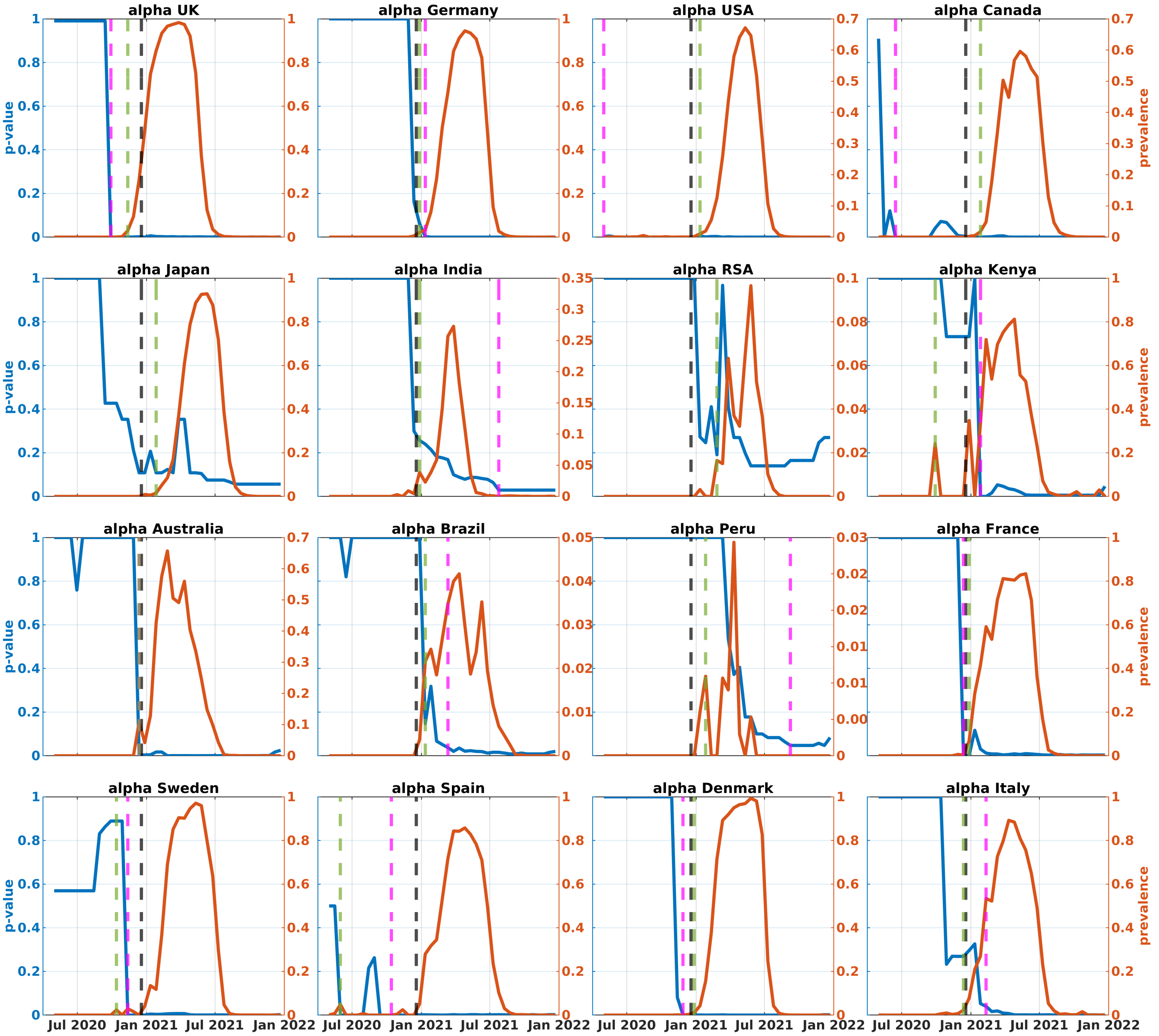

Supplementary Fig. 2: p-values (blue) and prevalences (red) of Alpha variant in the analyzed countries (complete dataset). Black, green, and magenta lines represent the times of VOC designation, achieving 1% prevalence, and becoming significantly dense, respectively.

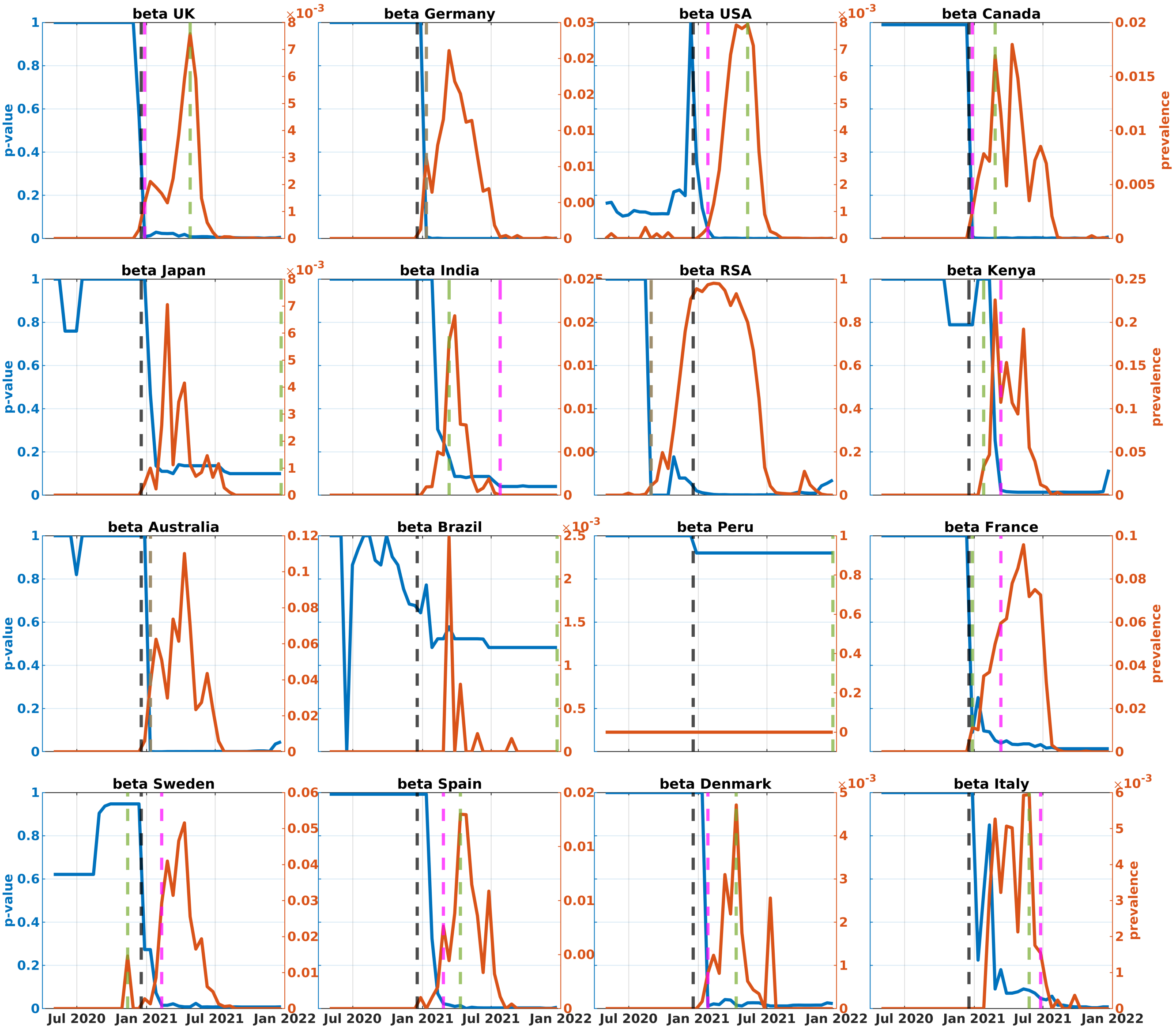

Supplementary Fig. 3: p-values (blue) and prevalences (red) of Beta variant in the analyzed countries (complete dataset). Black, green, and magenta lines represent the times of VOC designation, achieving 1% prevalence, and becoming significantly dense, respectively.

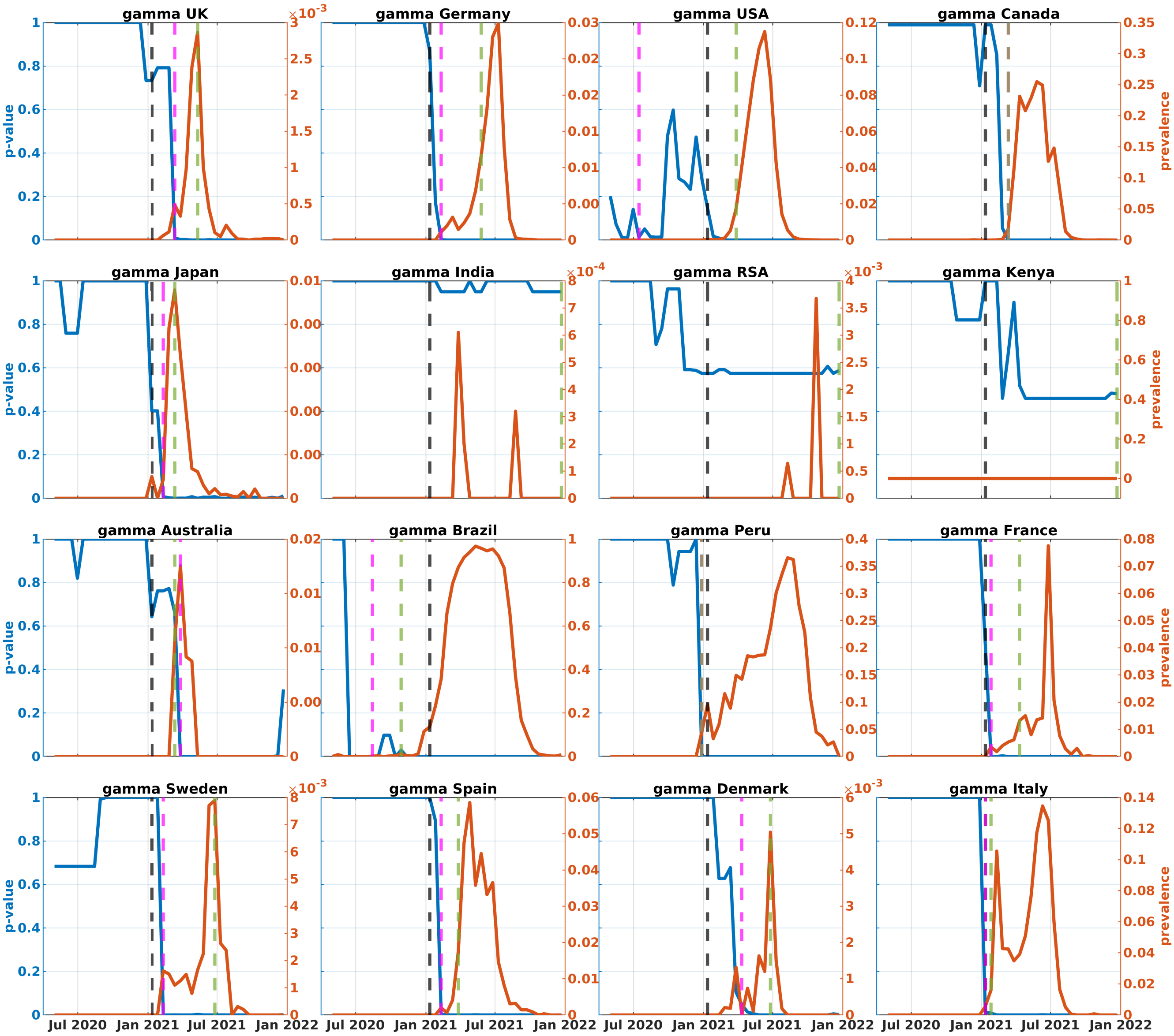

Supplementary Fig. 4: p-values (blue) and prevalences (red) of Gamma variant in the analyzed countries (complete dataset). Black, green, and magenta lines represent the times of VOC designation, achieving 1% prevalence, and becoming significantly dense, respectively.

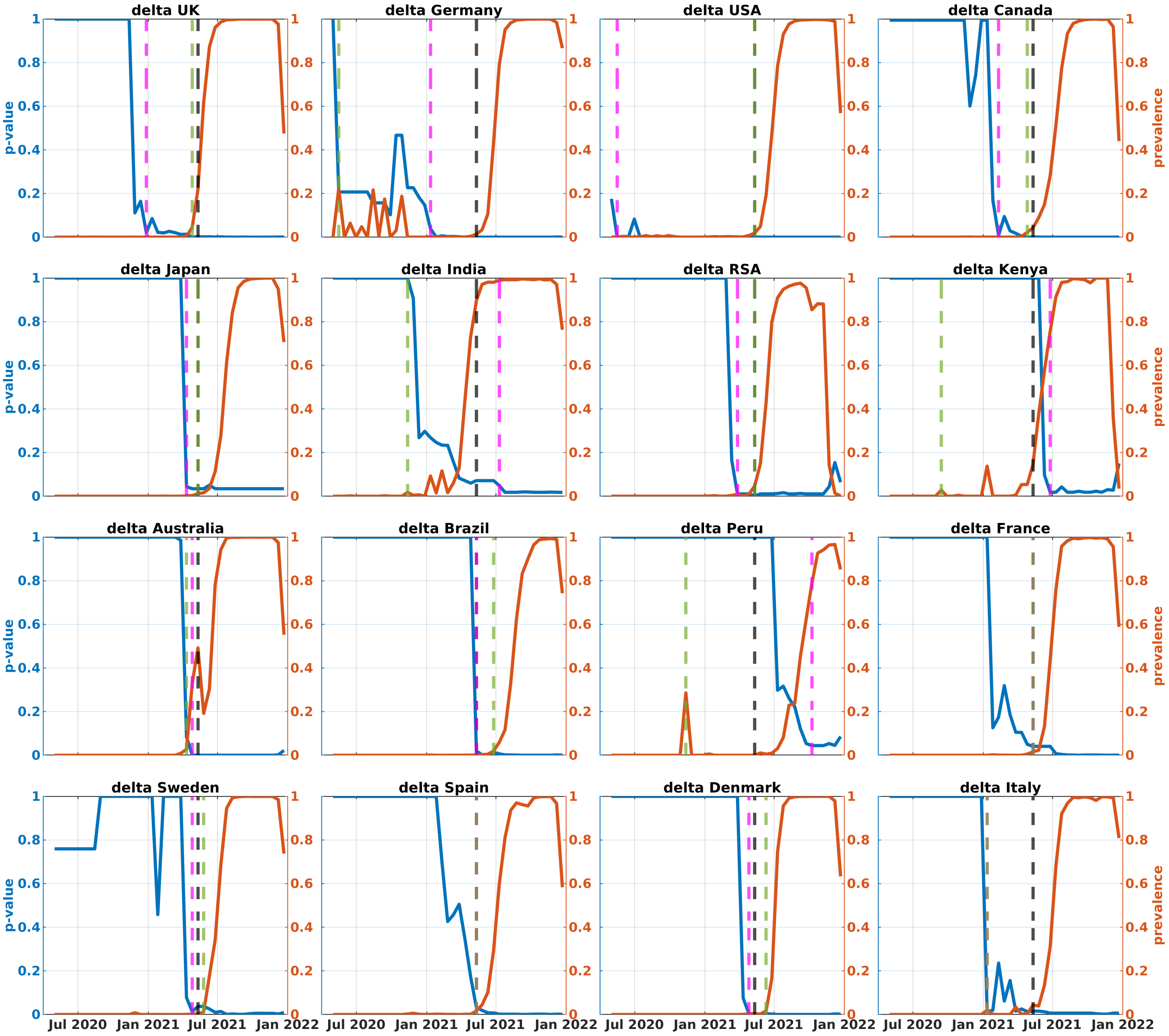

Supplementary Fig. 5: p-values (blue) and prevalences (red) of Delta variant in the analyzed countries (complete dataset). Black, green, and magenta lines represent the times of VOC designation, achieving 1% prevalence, and becoming significantly dense, respectively.

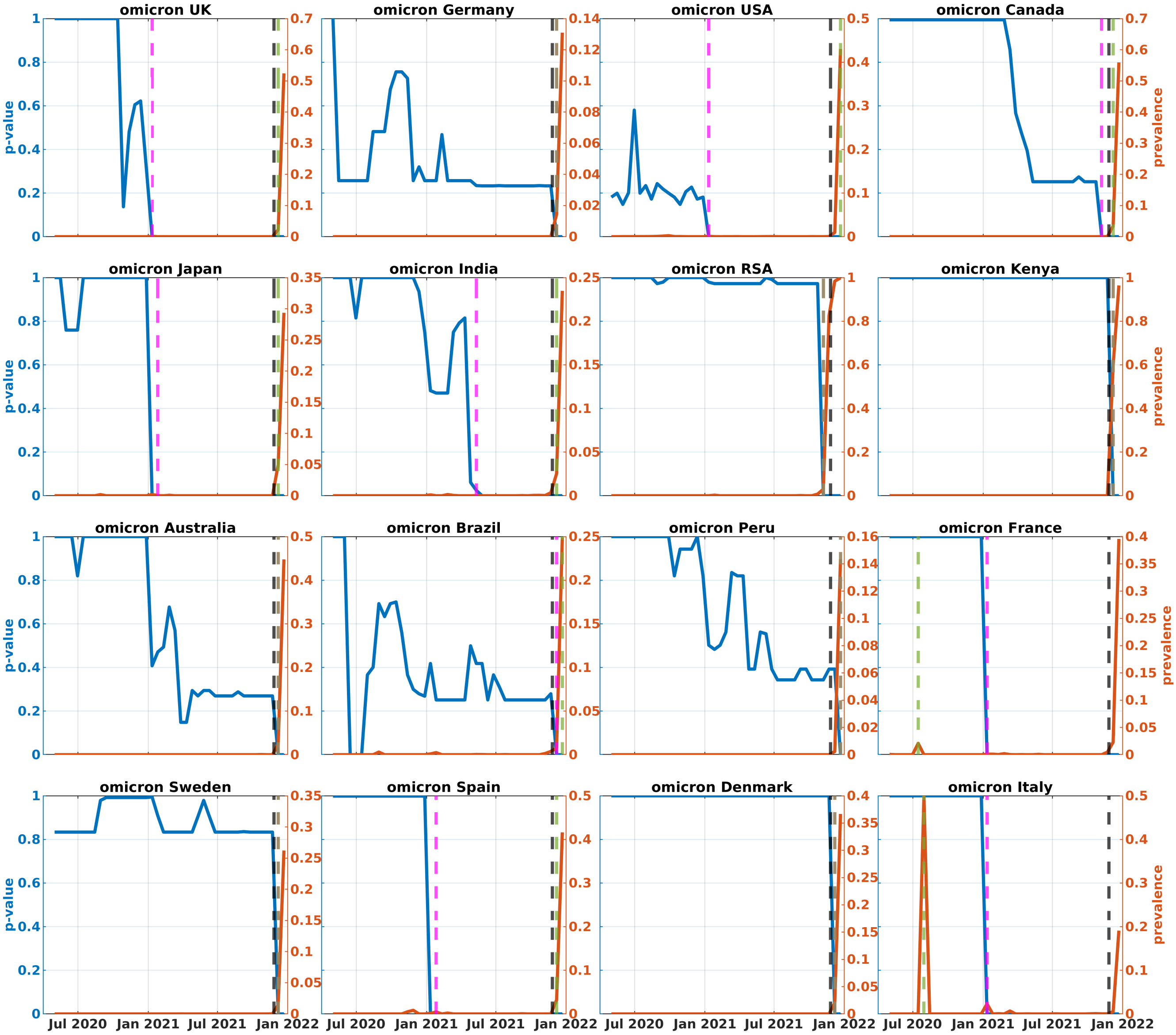

Supplementary Fig. 6: p-values (blue) and prevalences (red) of Omicron variant in the analyzed countries (complete dataset). Black, green, and magenta lines represent the times of VOC designation, achieving 1% prevalence, and becoming significantly dense, respectively.

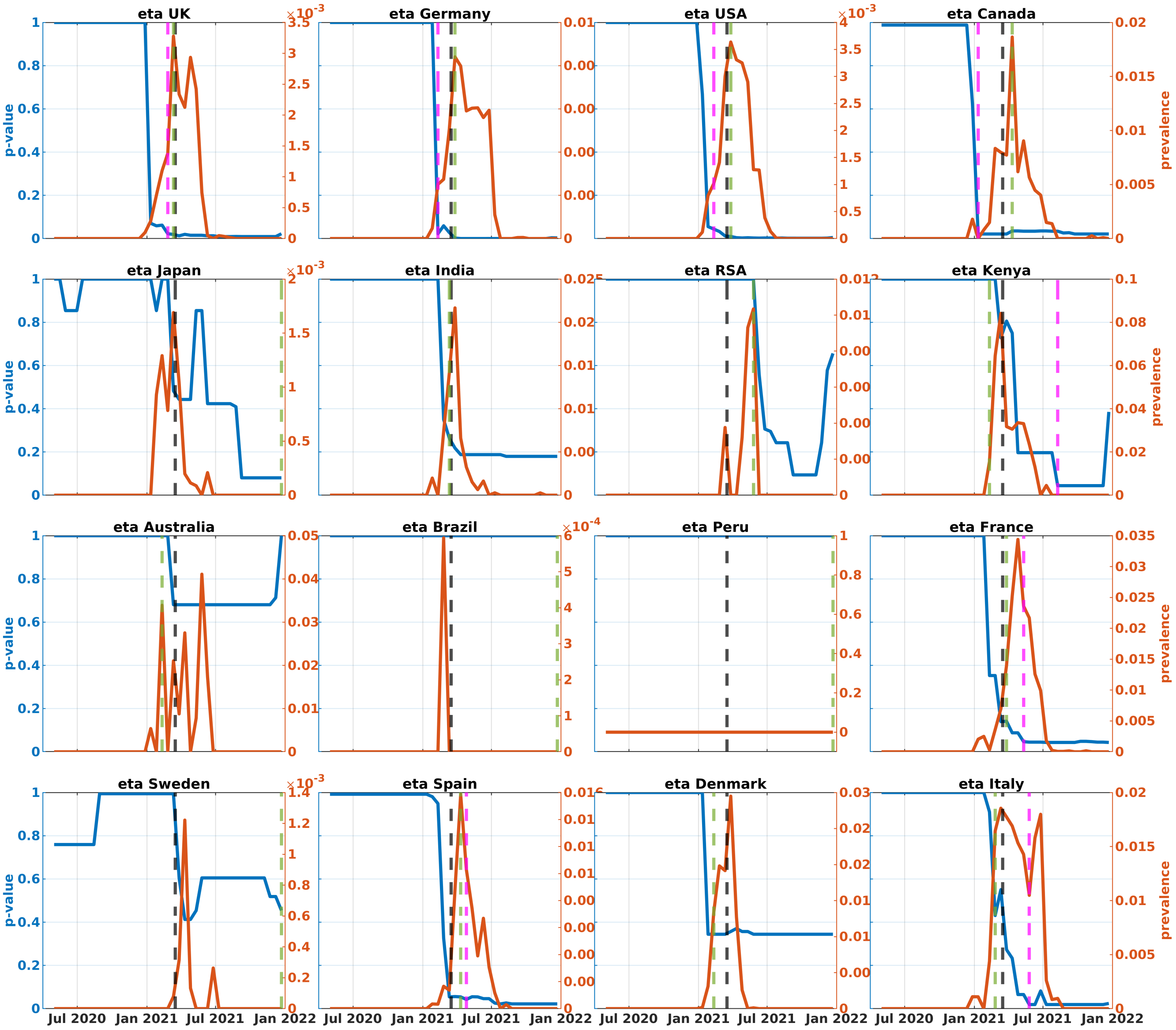

Supplementary Fig. 7: p-values (blue) and prevalences (red) of Eta variant in the analyzed countries (complete dataset). Black, green, and magenta lines represent the times of VOC designation, achieving 1% prevalence, and becoming significantly dense, respectively.

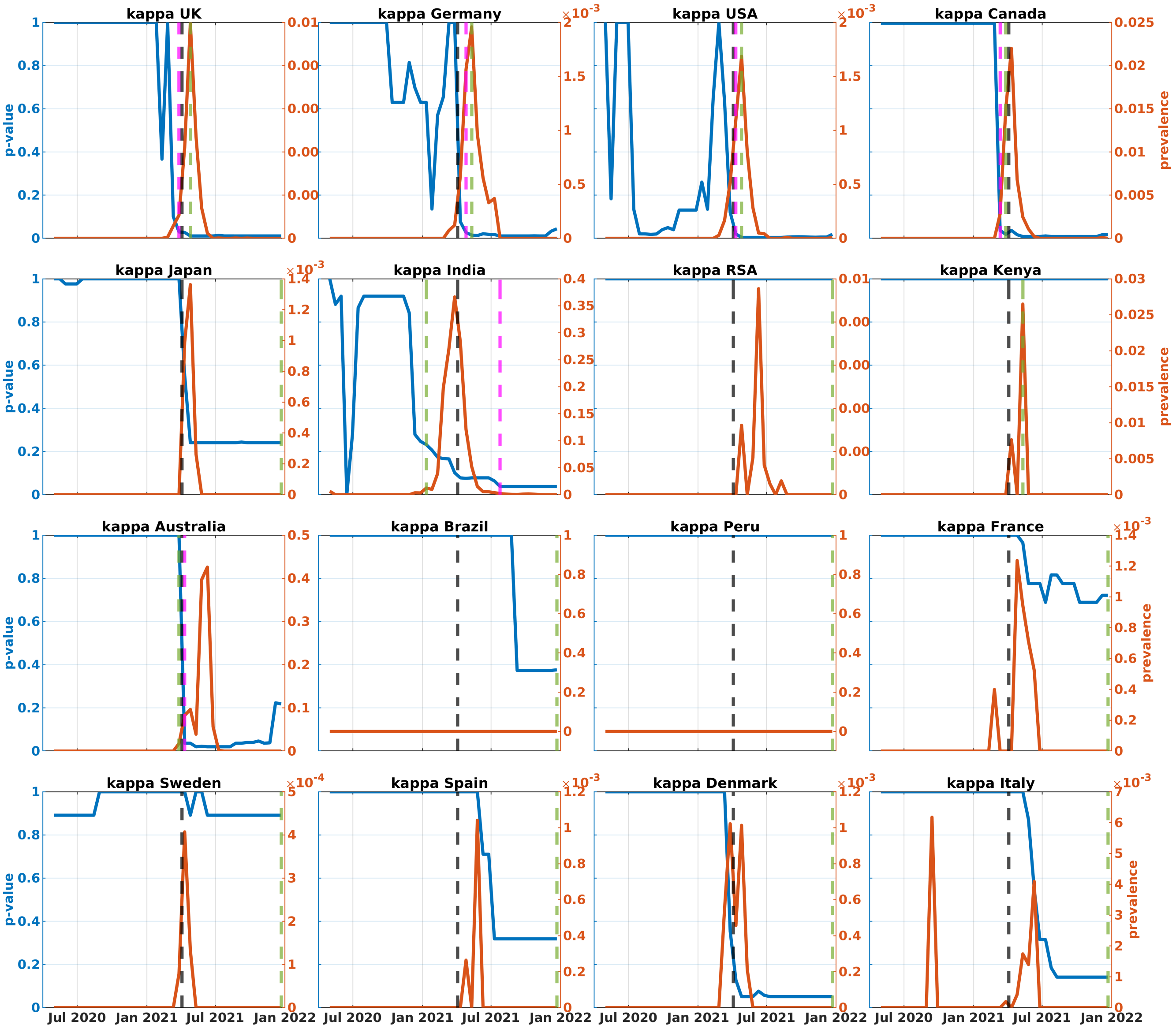

Supplementary Fig. 8: p-values (blue) and prevalences (red) of Kappa variant in the analyzed countries (complete dataset). Black, green, and magenta lines represent the times of VOC designation, achieving 1% prevalence, and becoming significantly dense, respectively.

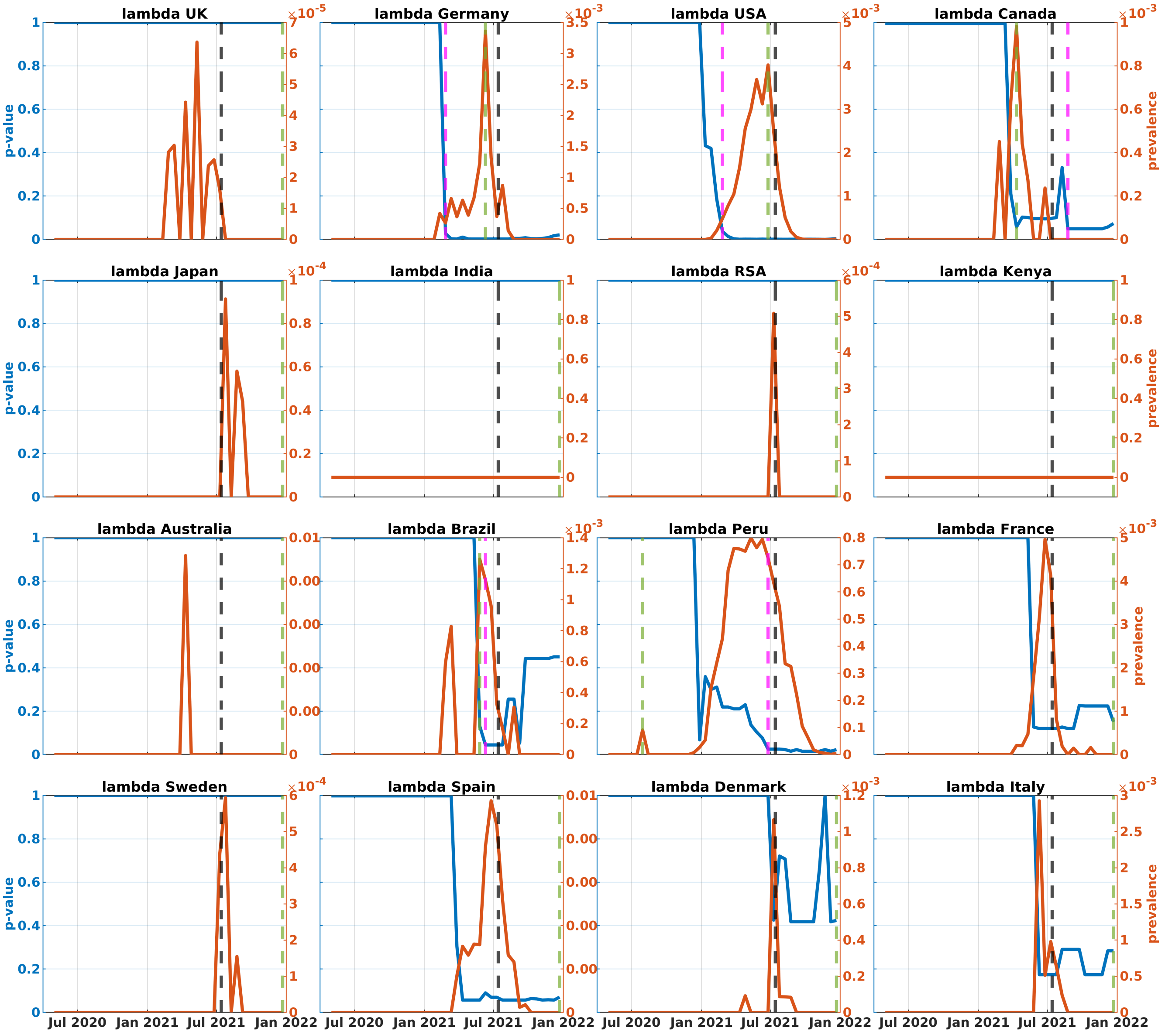

Supplementary Fig. 9: p-values (blue) and prevalences (red) of Lambda variant in the analyzed countries (complete dataset). Black, green, and magenta lines represent the times of VOC designation, achieving 1% prevalence, and becoming significantly dense, respectively.

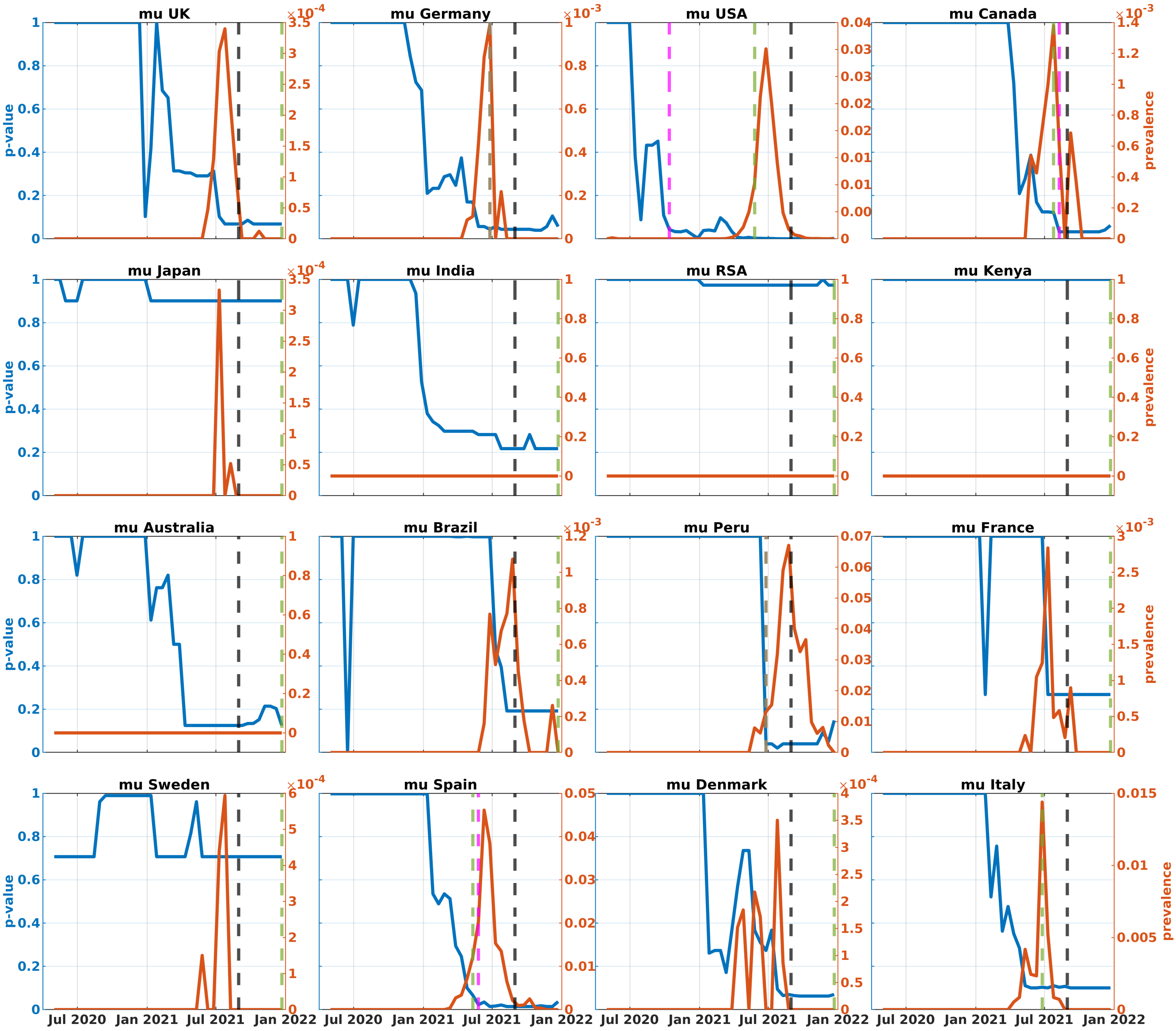

Supplementary Fig. 10: p-values (blue) and prevalences (red) of Mu variant in the analyzed countries (complete dataset). Black, green, and magenta lines represent the times of VOC designation, achieving 1% prevalence, and becoming significantly dense, respectively.

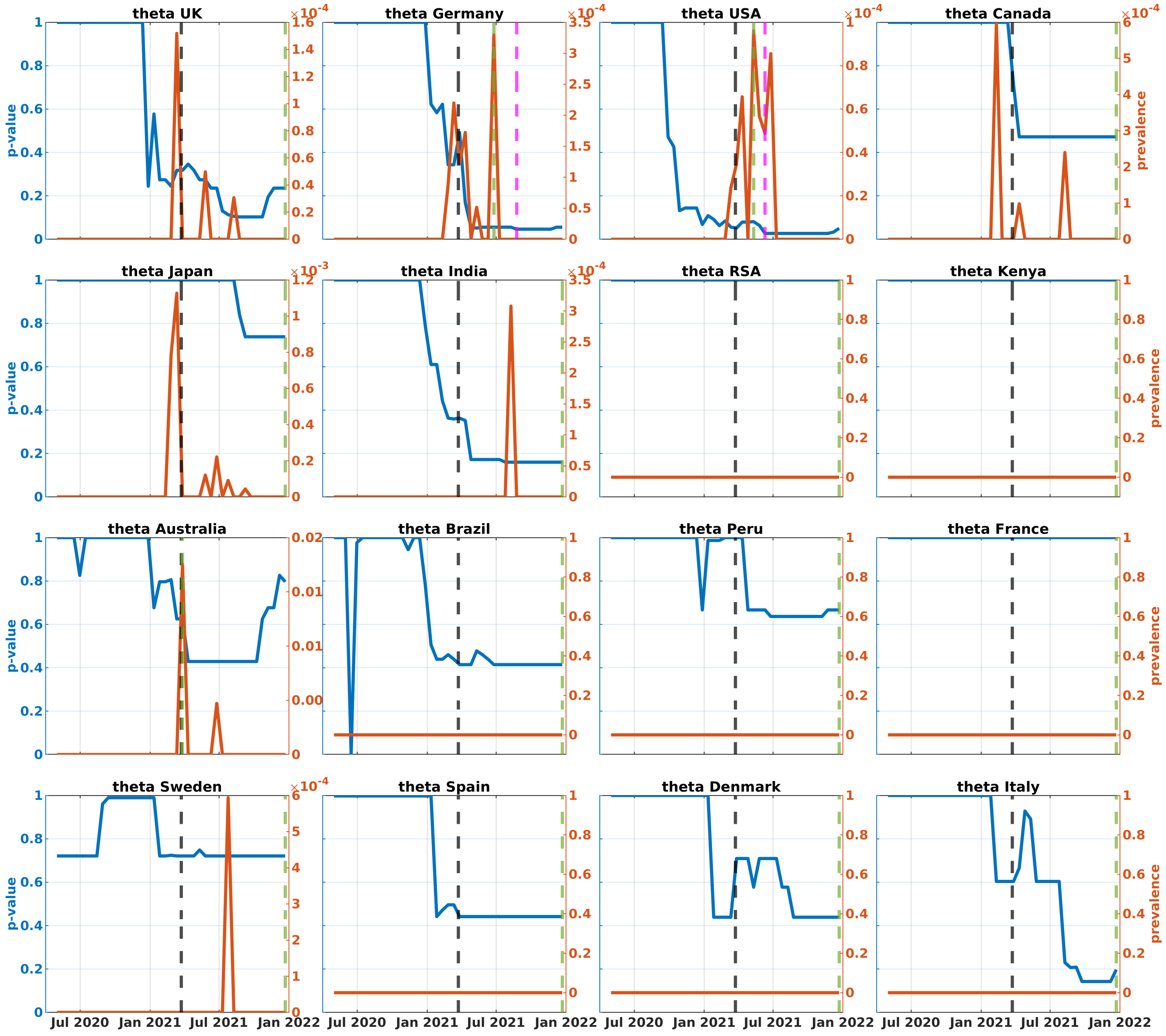

Supplementary Fig. 11: p-values (blue) and prevalences (red) of Theta variant in the analyzed countries (complete dataset). Black, green, and magenta lines represent the times of VOC designation, achieving 1% prevalence, and becoming significantly dense, respectively.

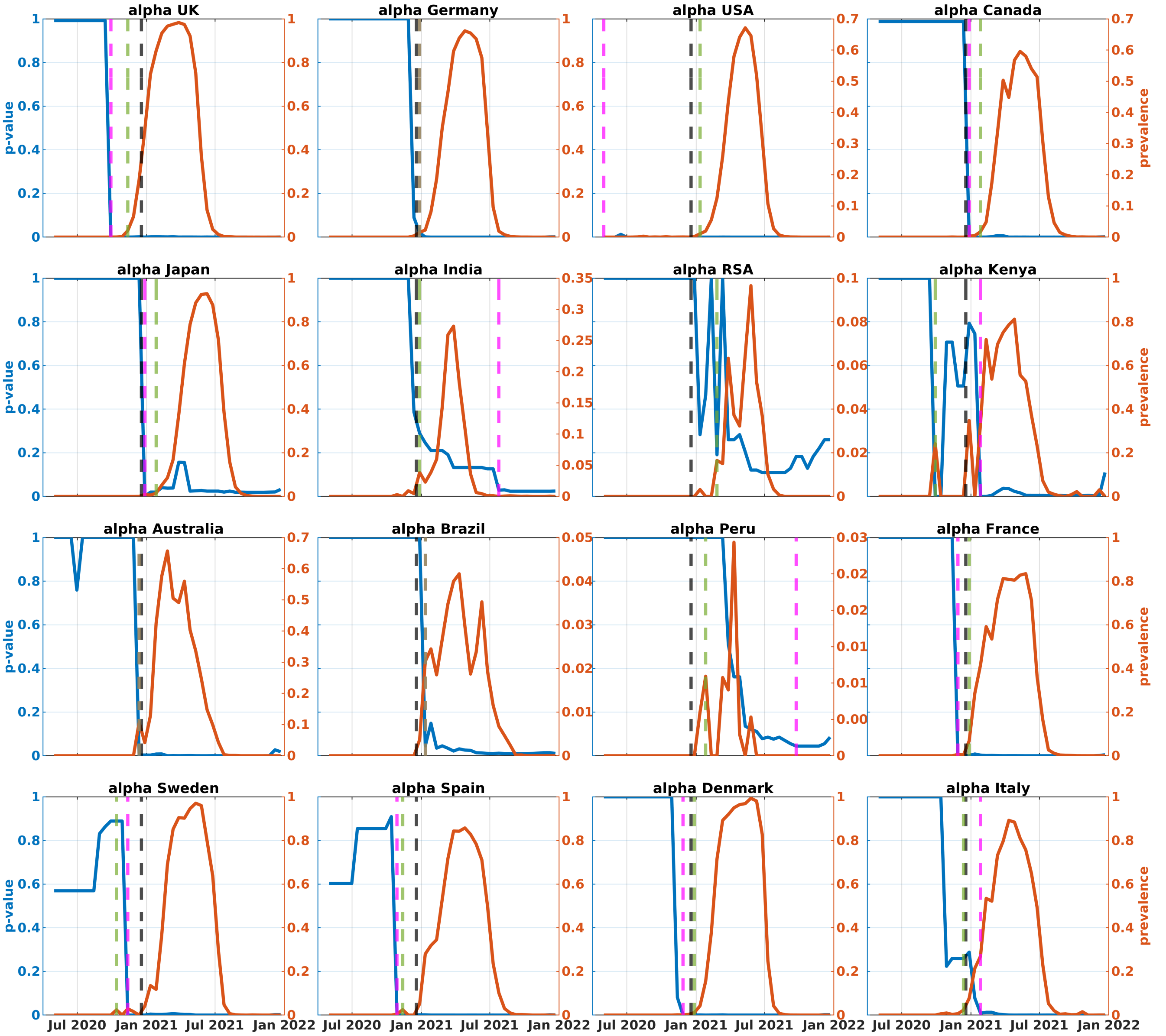

Supplementary Fig. 12: p-values (blue) and prevalences (red) of Alpha variant in the analyzed countries (first truncated dataset). Black, green, and magenta lines represent the times of VOC designation, achieving 1% prevalence, and becoming significantly dense, respectively.

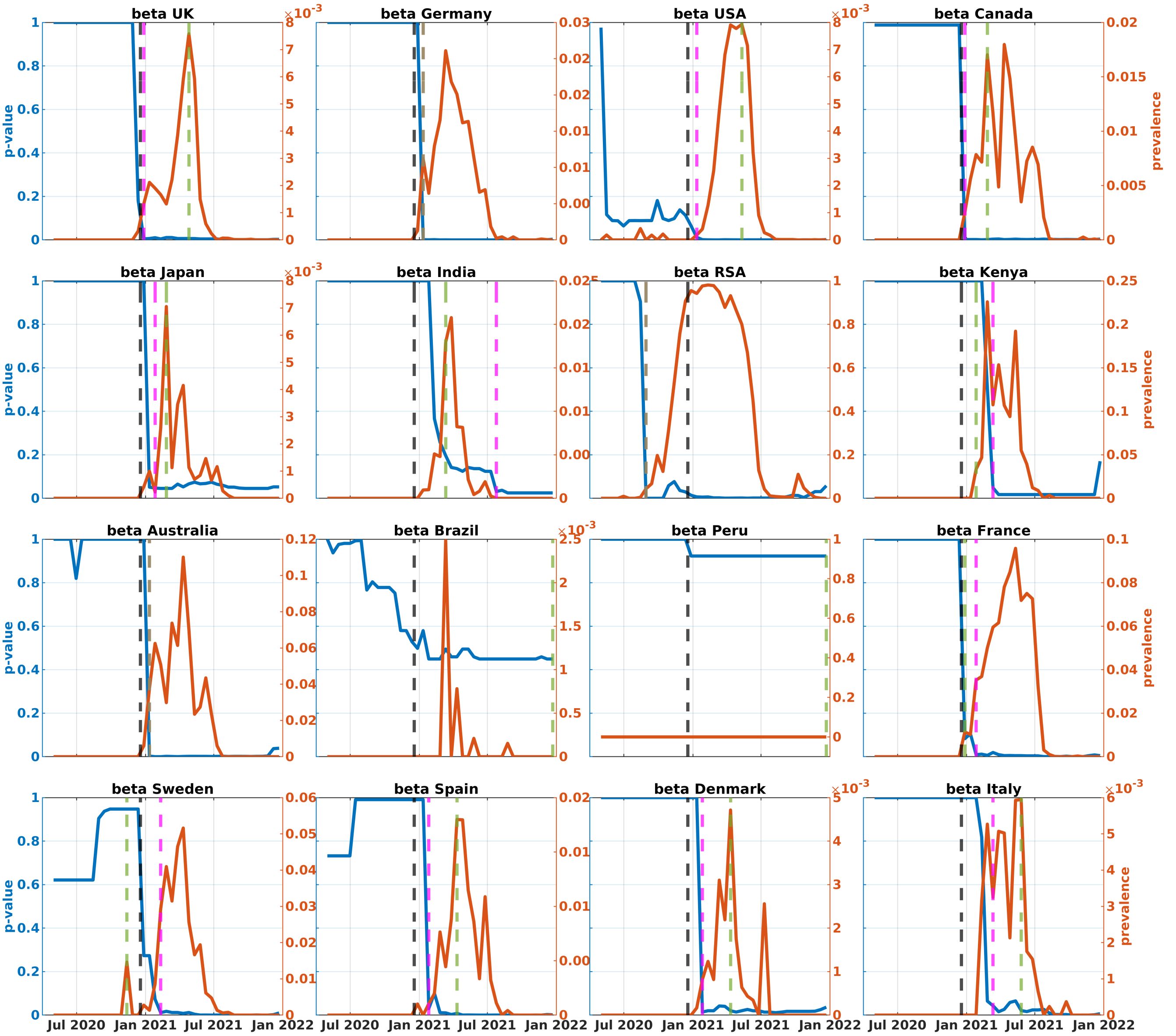

Supplementary Fig. 13: p-values (blue) and prevalences (red) of Beta variant in the analyzed countries (first truncated dataset). Black, green, and magenta lines represent the times of VOC designation, achieving 1% prevalence, and becoming significantly dense, respectively.

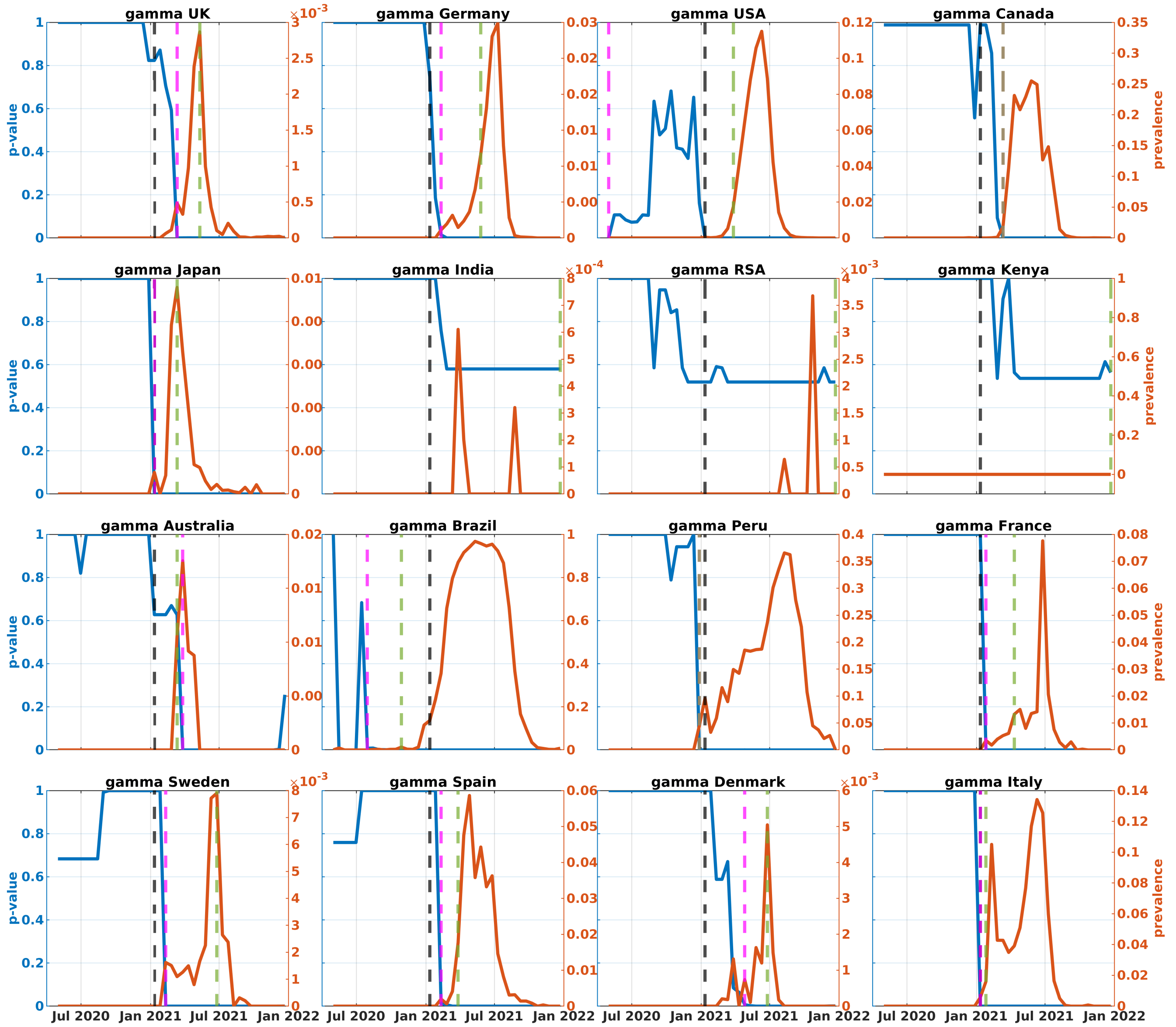

Supplementary Fig. 14: *p*-values (blue) and prevalences (red) of Gamma variant in the analyzed countries (first truncated dataset). Black, green, and magenta lines represent the times of VOC designation, achieving 1% prevalence, and becoming significantly dense, respectively.

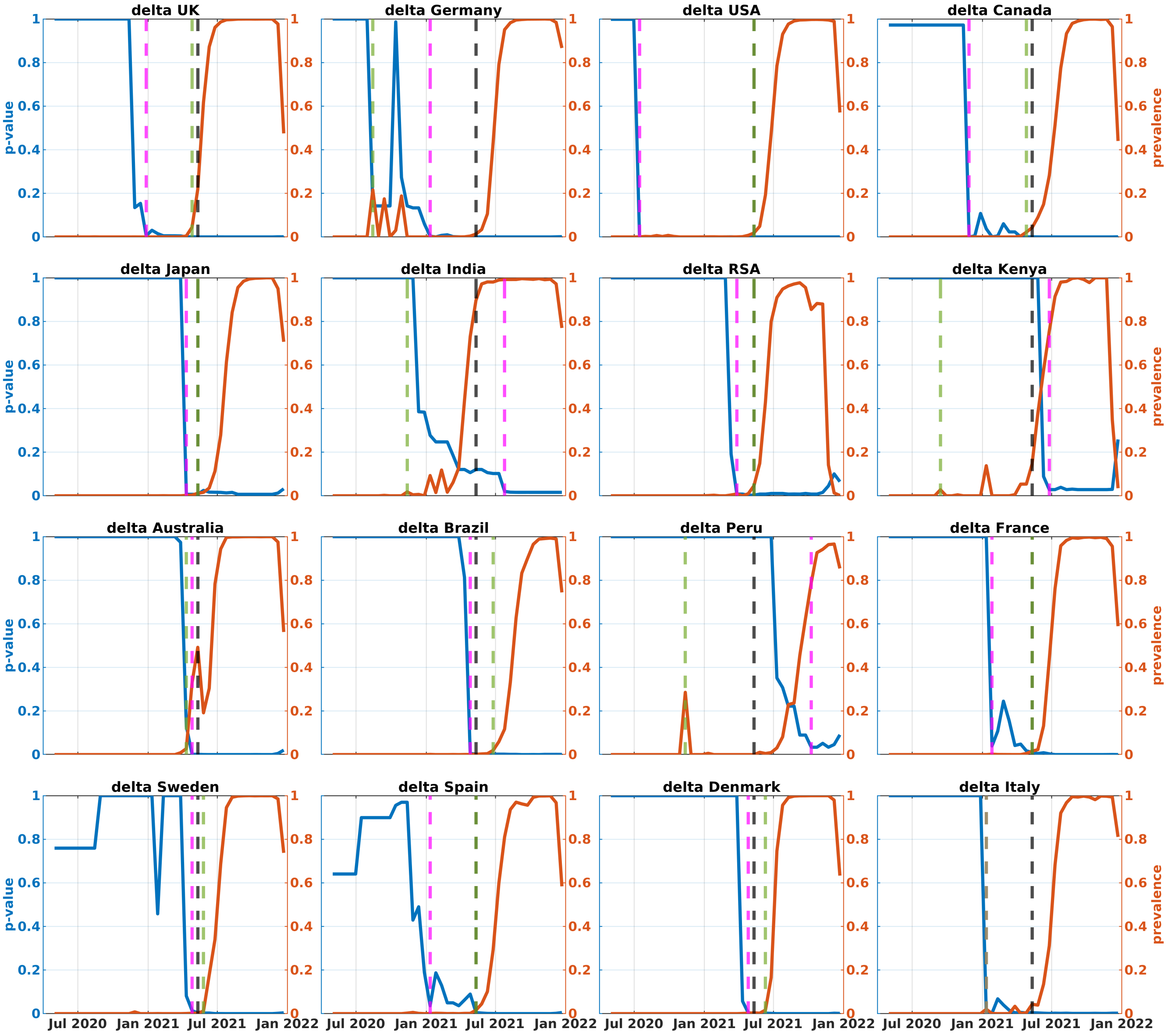

Supplementary Fig. 15: p-values (blue) and prevalences (red) of Delta variant in the analyzed countries (first truncated dataset). Black, green, and magenta lines represent the times of VOC designation, achieving 1% prevalence, and becoming significantly dense, respectively.

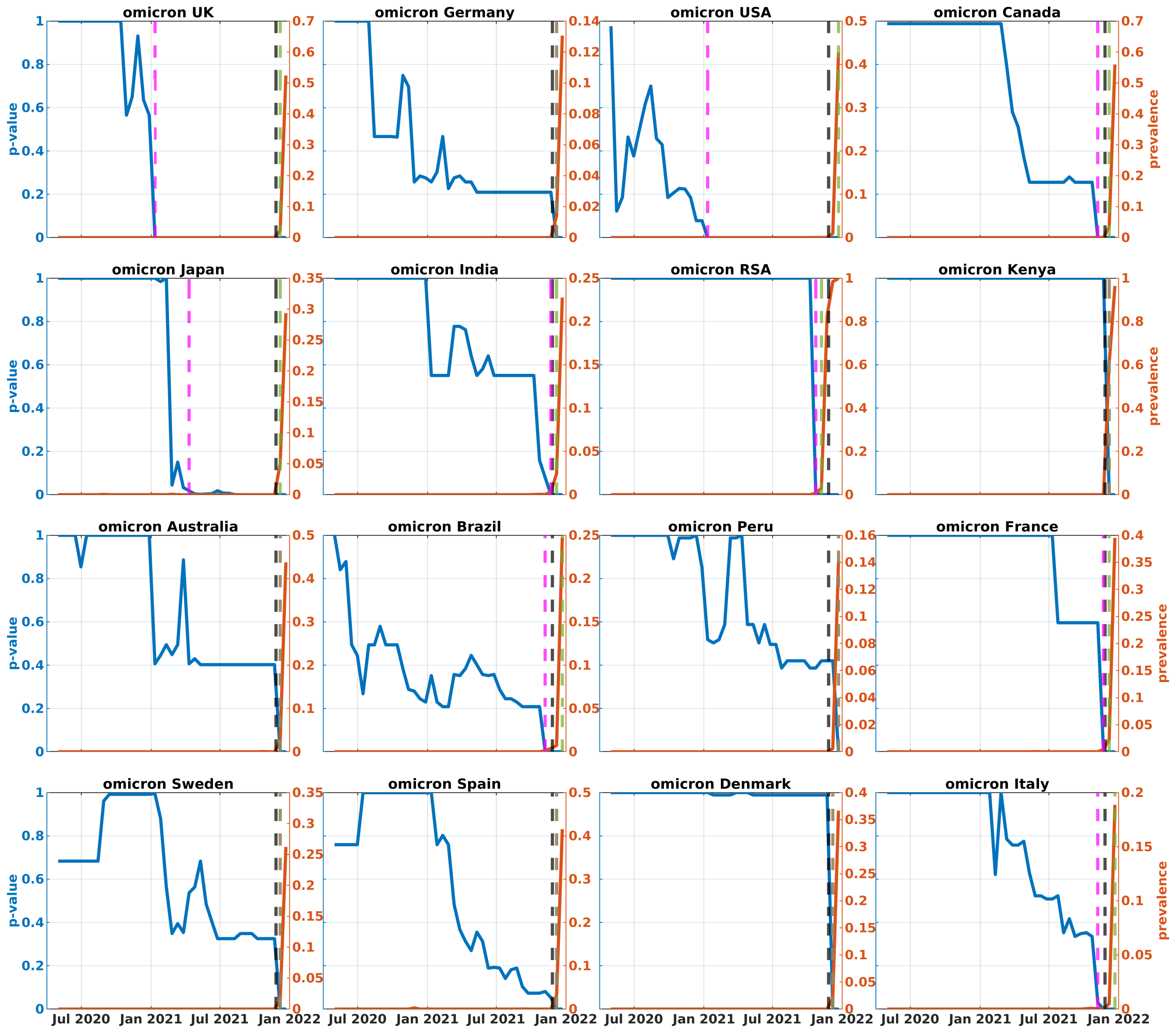

Supplementary Fig. 16: p-values (blue) and prevalences (red) of Omicron variant in the analyzed countries (first truncated dataset). Black, green, and magenta lines represent the times of VOC designation, achieving 1% prevalence, and becoming significantly dense, respectively.

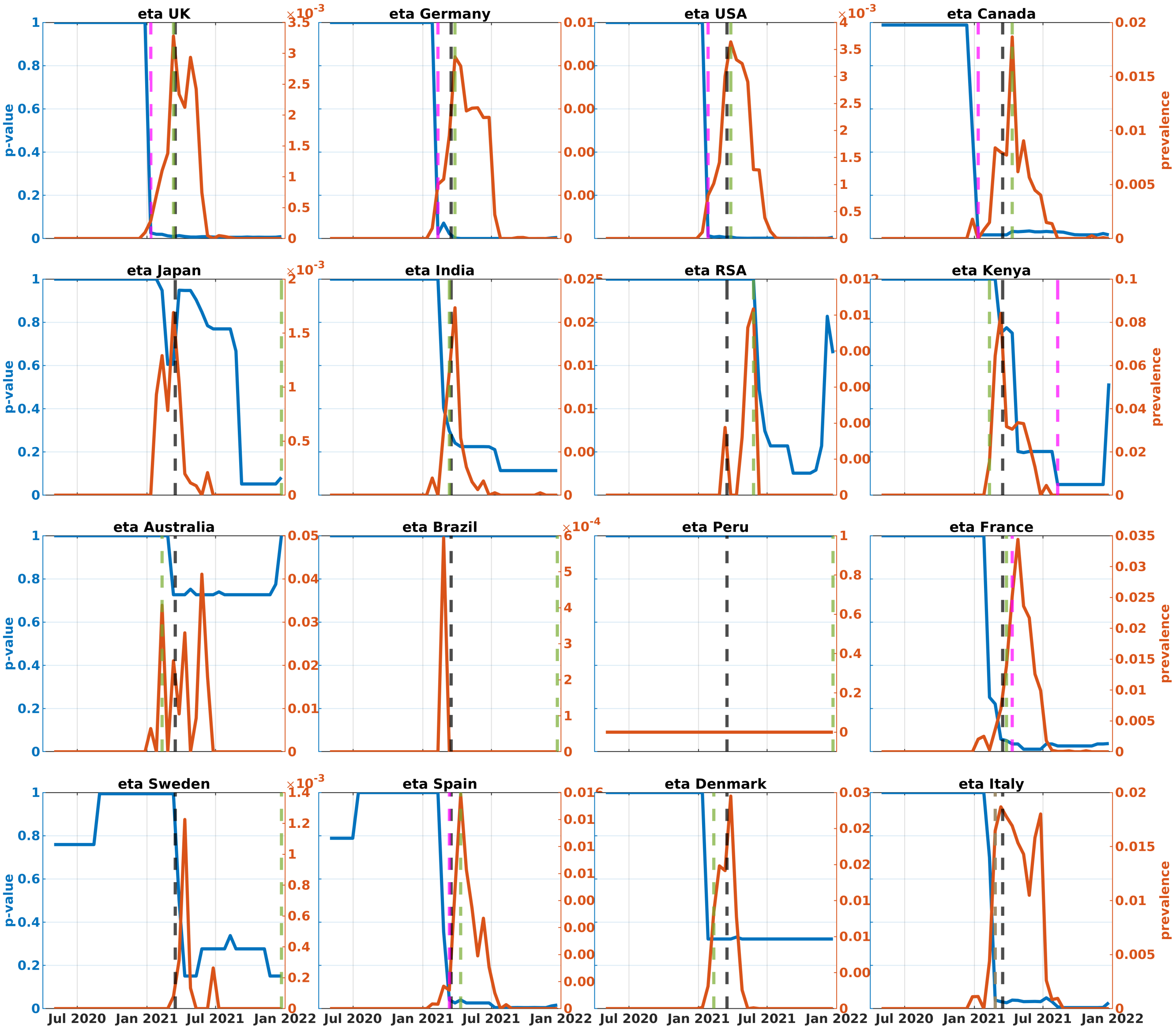

Supplementary Fig. 17: p-values (blue) and prevalences (red) of Eta variant in the analyzed countries (first truncated dataset). Black, green, and magenta lines represent the times of VOC designation, achieving 1% prevalence, and becoming significantly dense, respectively.

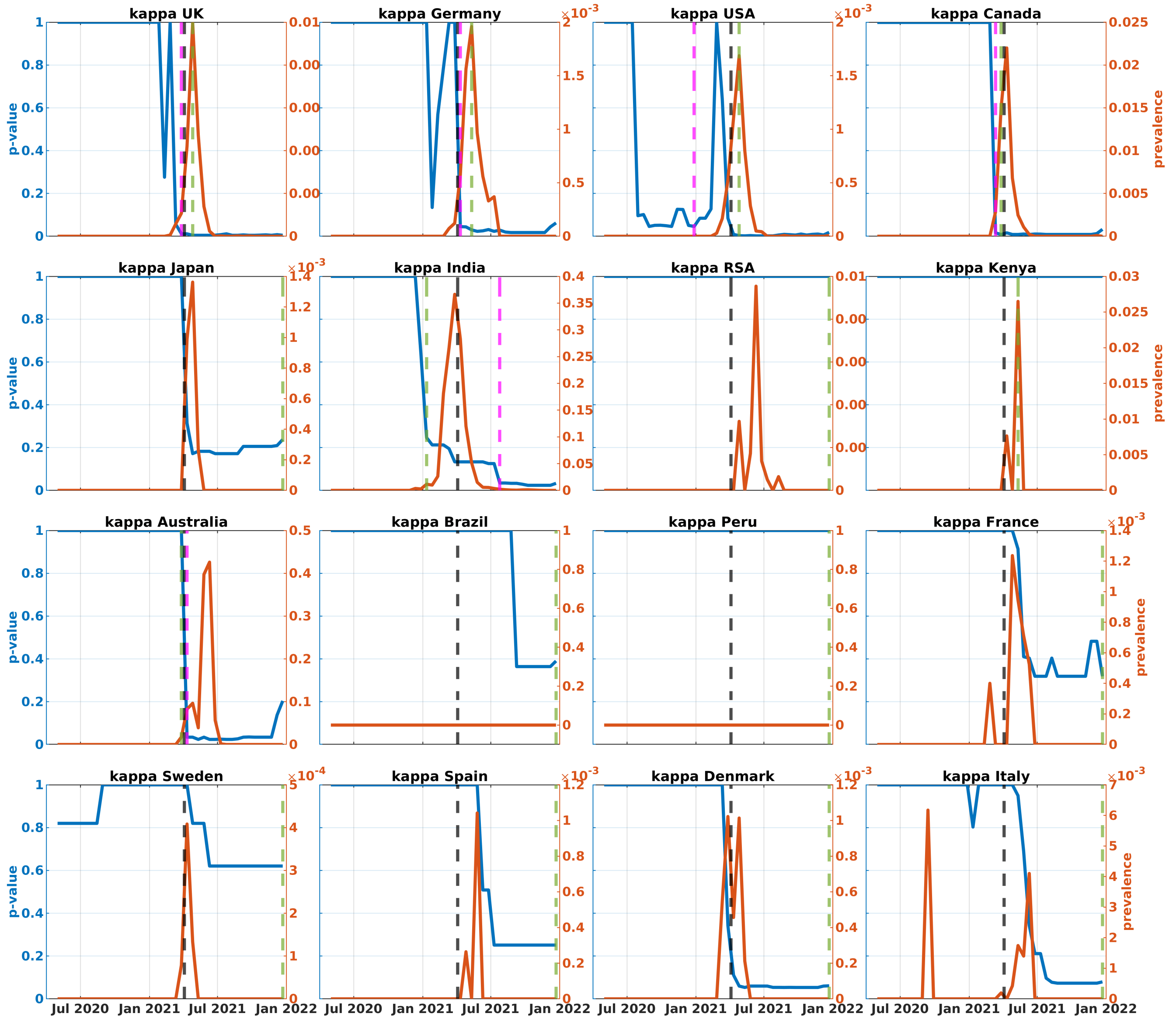

Supplementary Fig. 18: p-values (blue) and prevalences (red) of Kappa variant in the analyzed countries (first truncated dataset). Black, green, and magenta lines represent the times of VOC designation, achieving 1% prevalence, and becoming significantly dense, respectively.

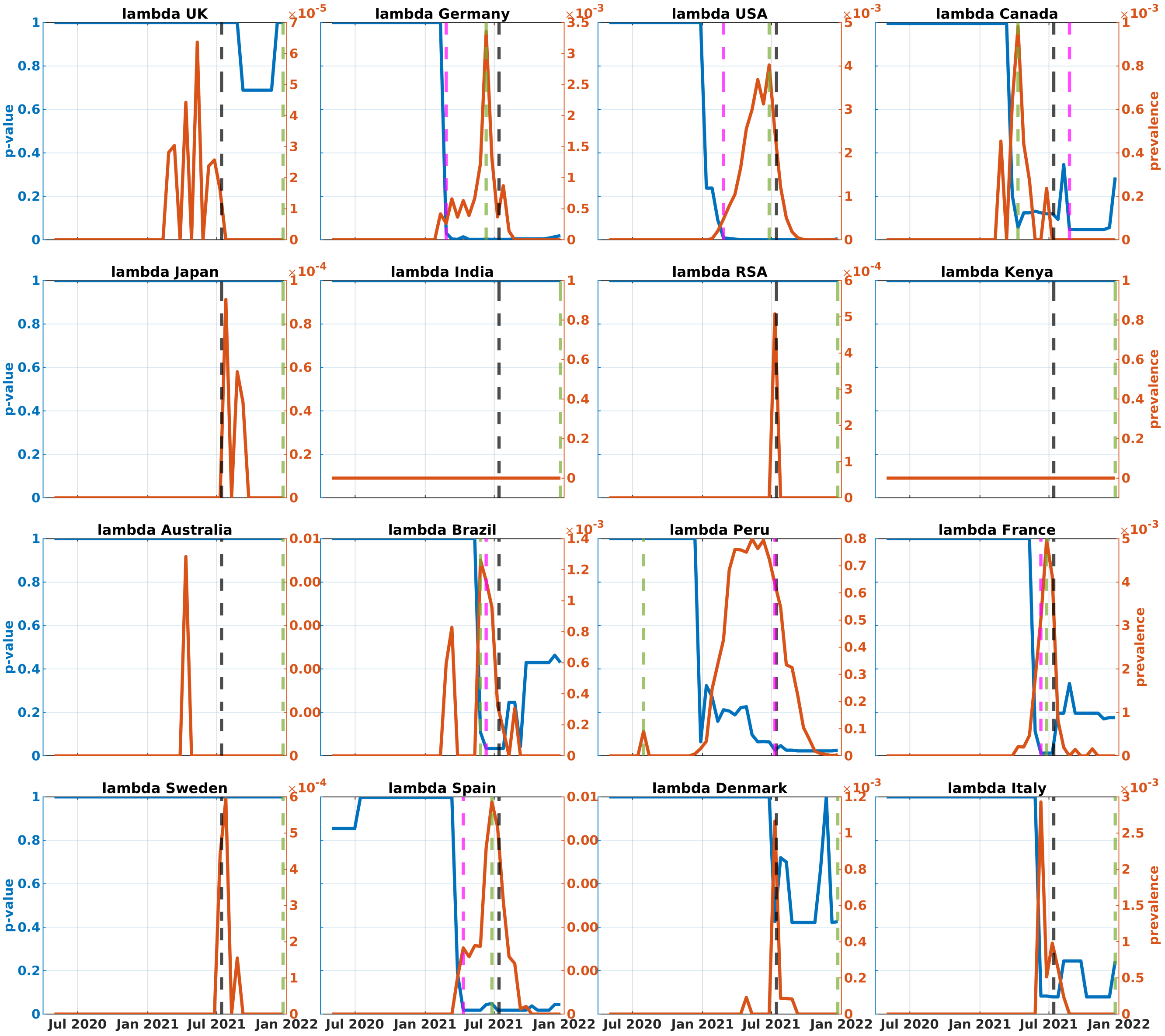

Supplementary Fig. 19: p-values (blue) and prevalences (red) of Lambda variant in the analyzed countries (first truncated dataset). Black, green, and magenta lines represent the times of VOC designation, achieving 1% prevalence, and becoming significantly dense, respectively.

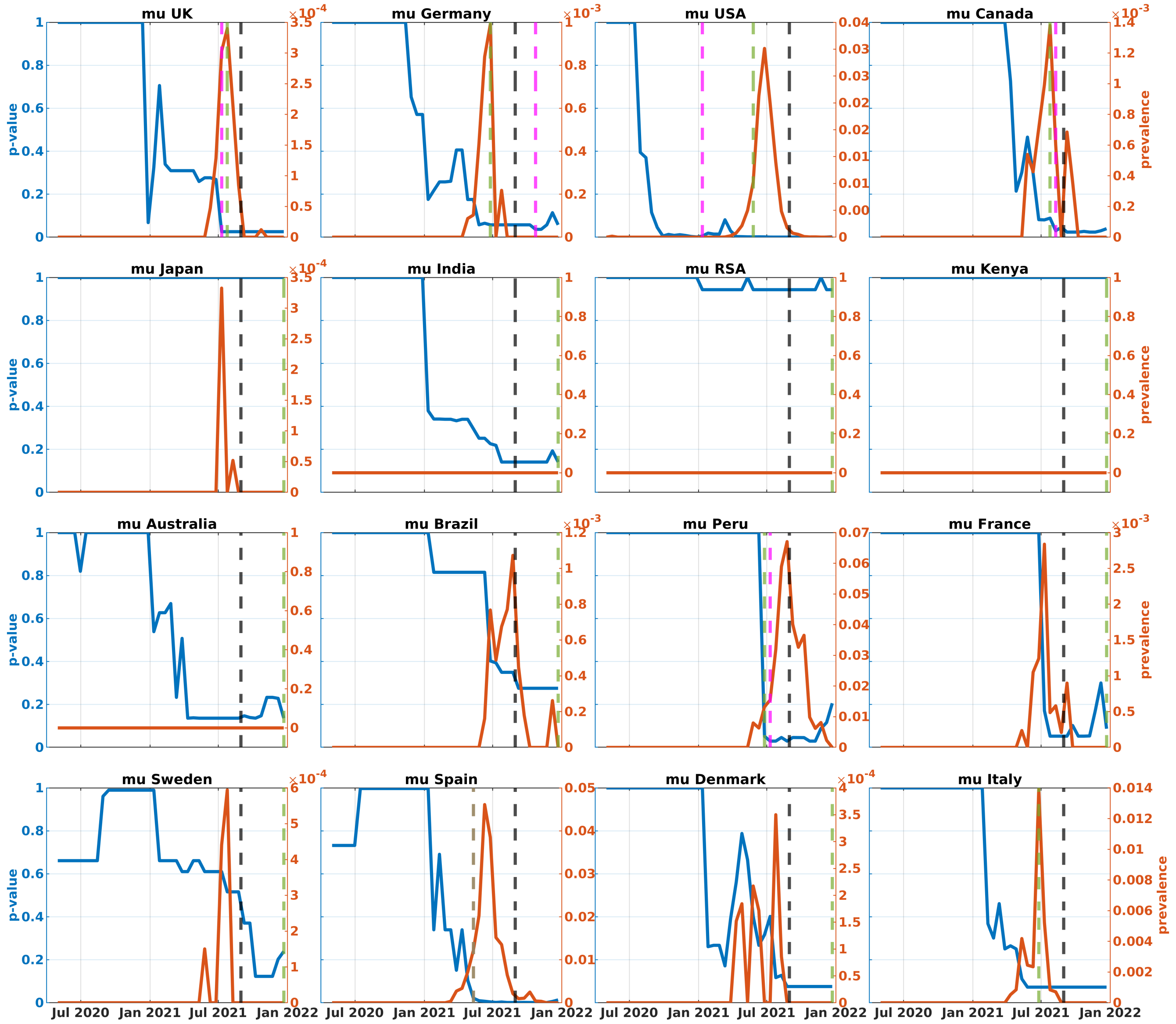

Supplementary Fig. 20: p-values (blue) and prevalences (red) of Mu variant in the analyzed countries (first truncated dataset). Black, green, and magenta lines represent the times of VOC designation, achieving 1% prevalence, and becoming significantly dense, respectively.

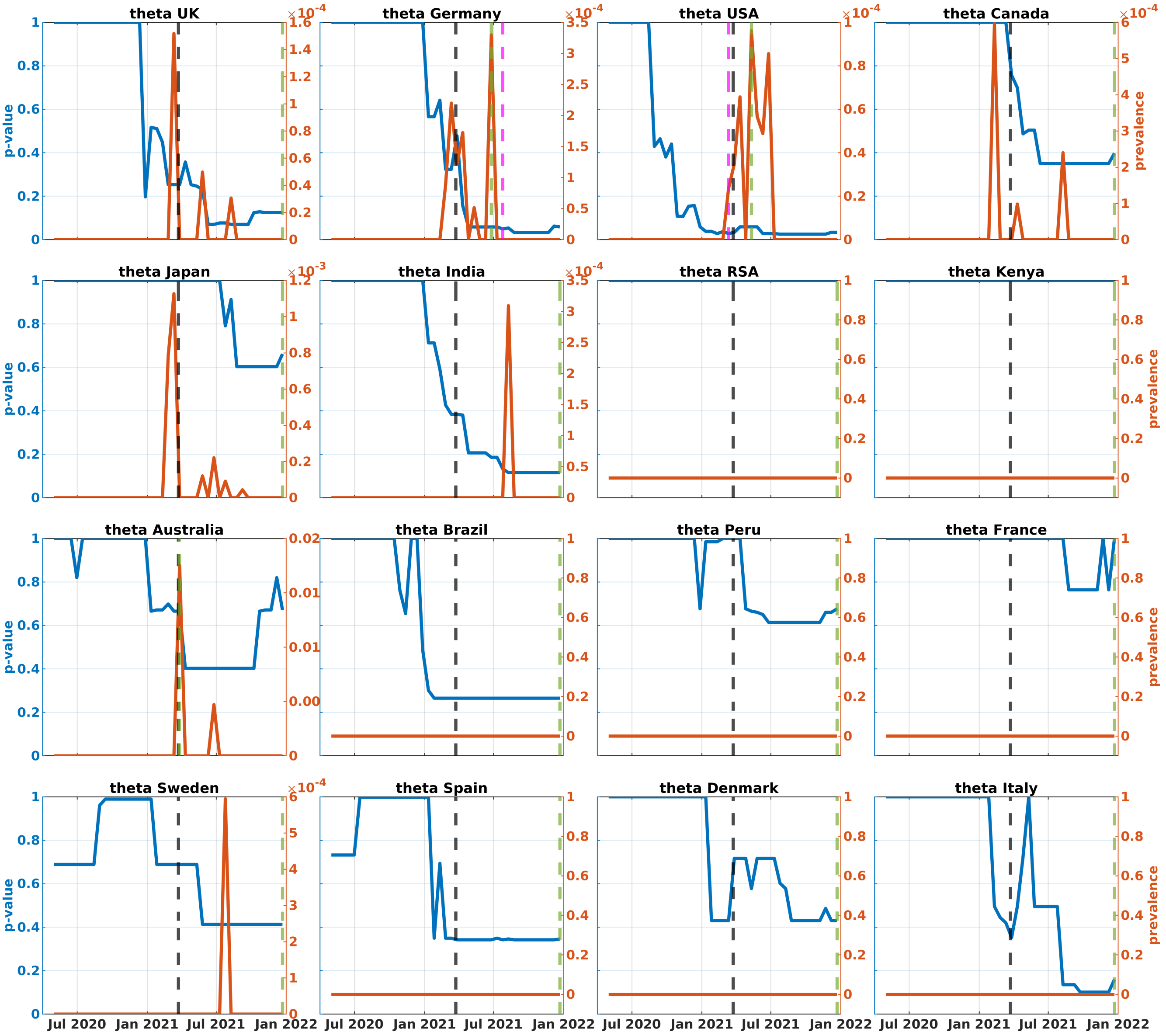

Supplementary Fig. 21: p-values (blue) and prevalences (red) of Theta variant in the analyzed countries (first truncated dataset). Black, green, and magenta lines represent the times of VOC designation, achieving 1% prevalence, and becoming significantly dense, respectively.

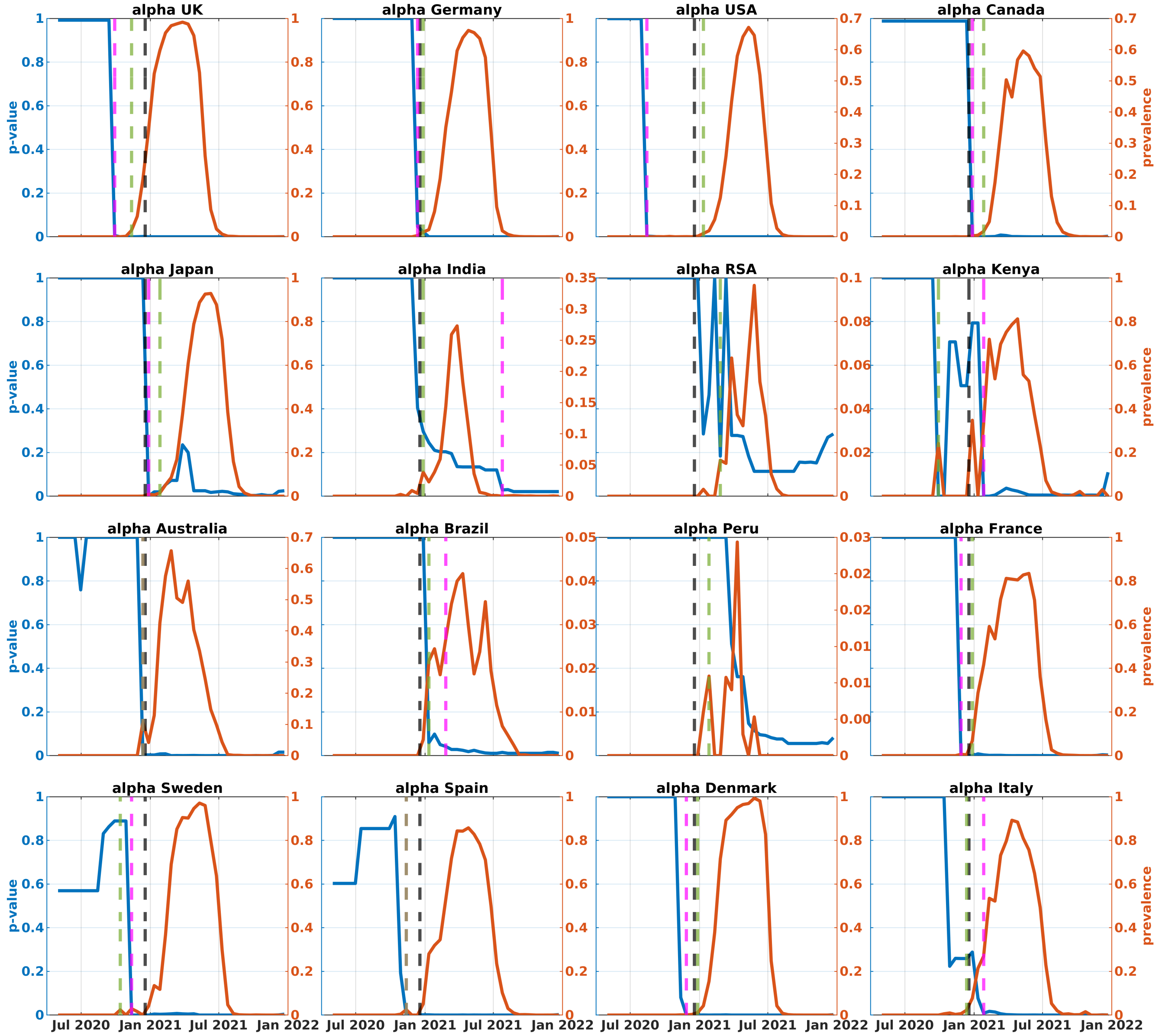

Supplementary Fig. 22: p-values (blue) and prevalences (red) of Alpha variant in the analyzed countries (second truncated dataset). Black, green, and magenta lines represent the times of VOC designation, achieving 1% prevalence, and becoming significantly dense, respectively.

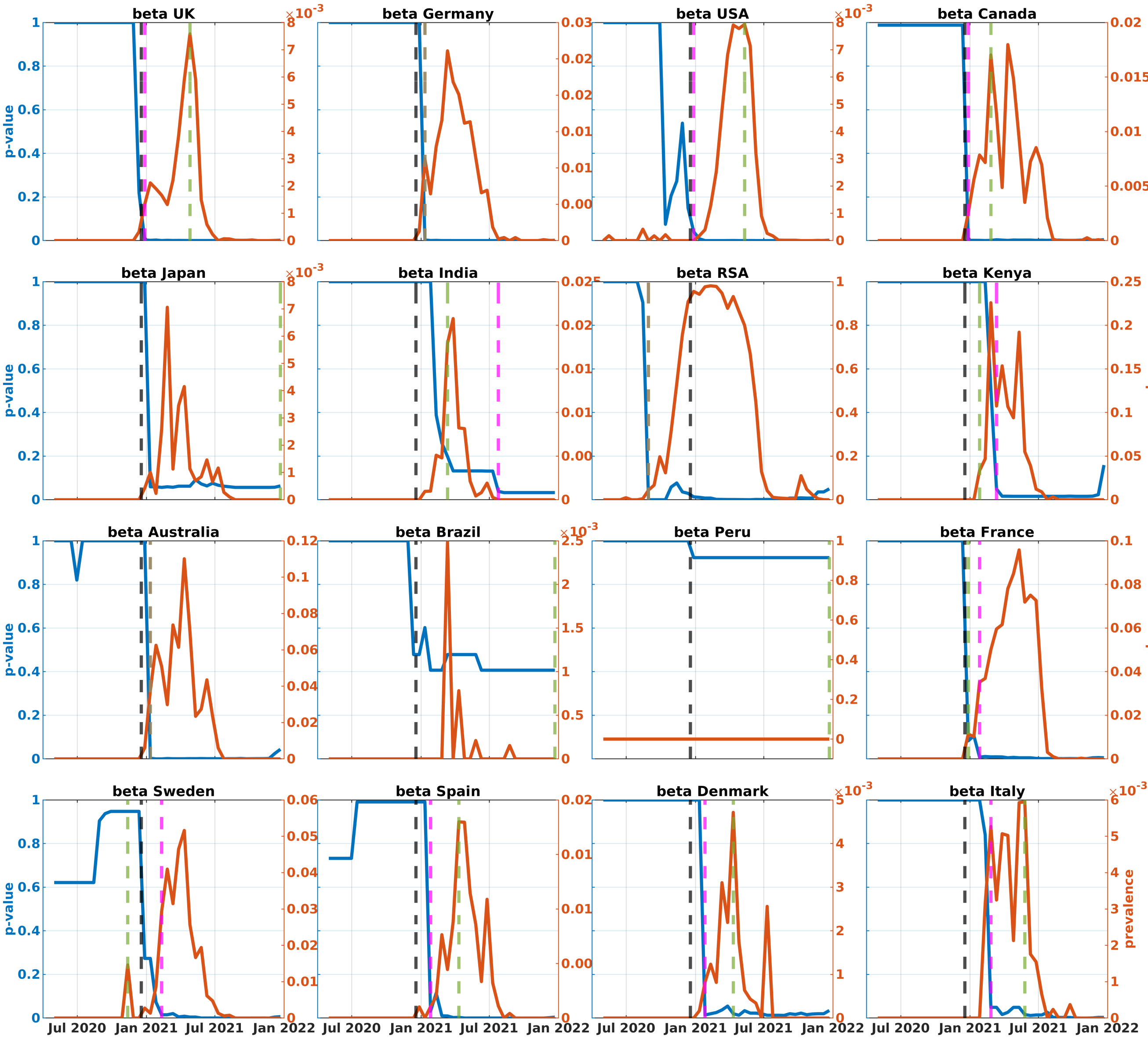

Supplementary Fig. 23: p-values (blue) and prevalences (red) of Beta variant in the analyzed countries (second truncated dataset). Black, green, and magenta lines represent the times of VOC designation, achieving 1% prevalence, and becoming significantly dense, respectively.

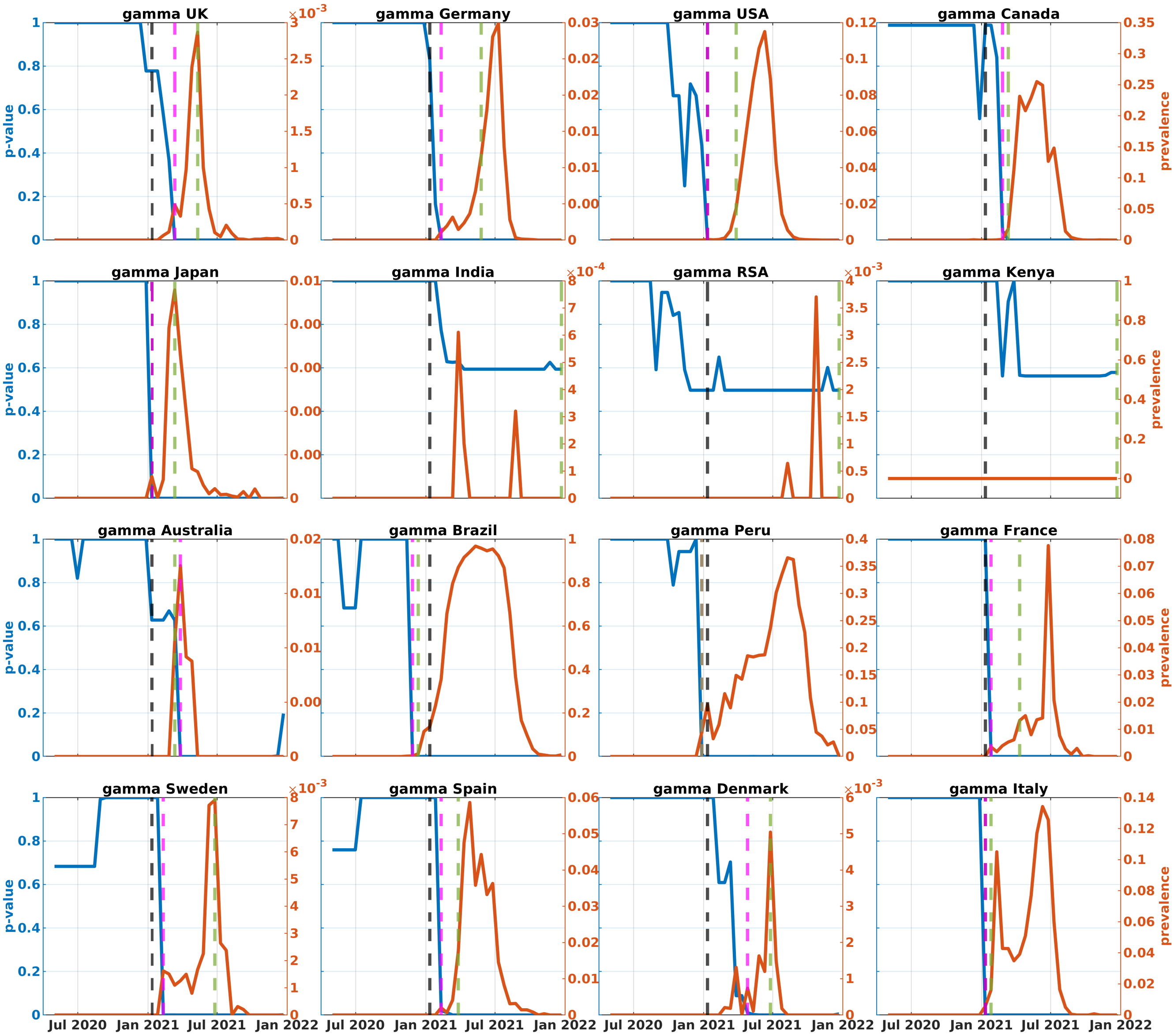

Supplementary Fig. 24: *p*-values (blue) and prevalences (red) of Gamma variant in the analyzed countries (second truncated dataset). Black, green, and magenta lines represent the times of VOC designation, achieving 1% prevalence, and becoming significantly dense, respectively.

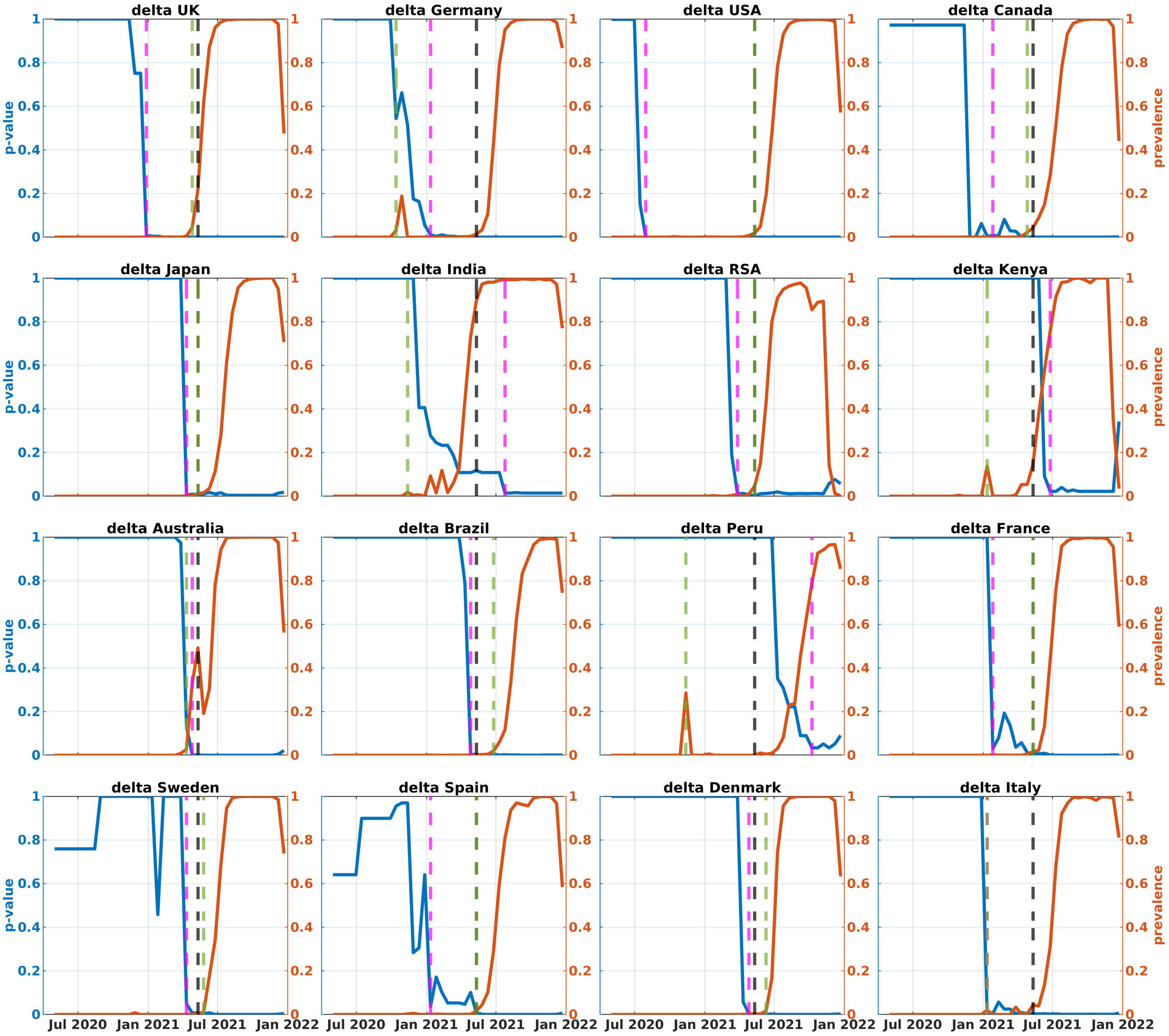

Supplementary Fig. 25: p-values (blue) and prevalences (red) of Delta variant in the analyzed countries (second truncated dataset). Black, green, and magenta lines represent the times of VOC designation, achieving 1% prevalence, and becoming significantly dense, respectively.

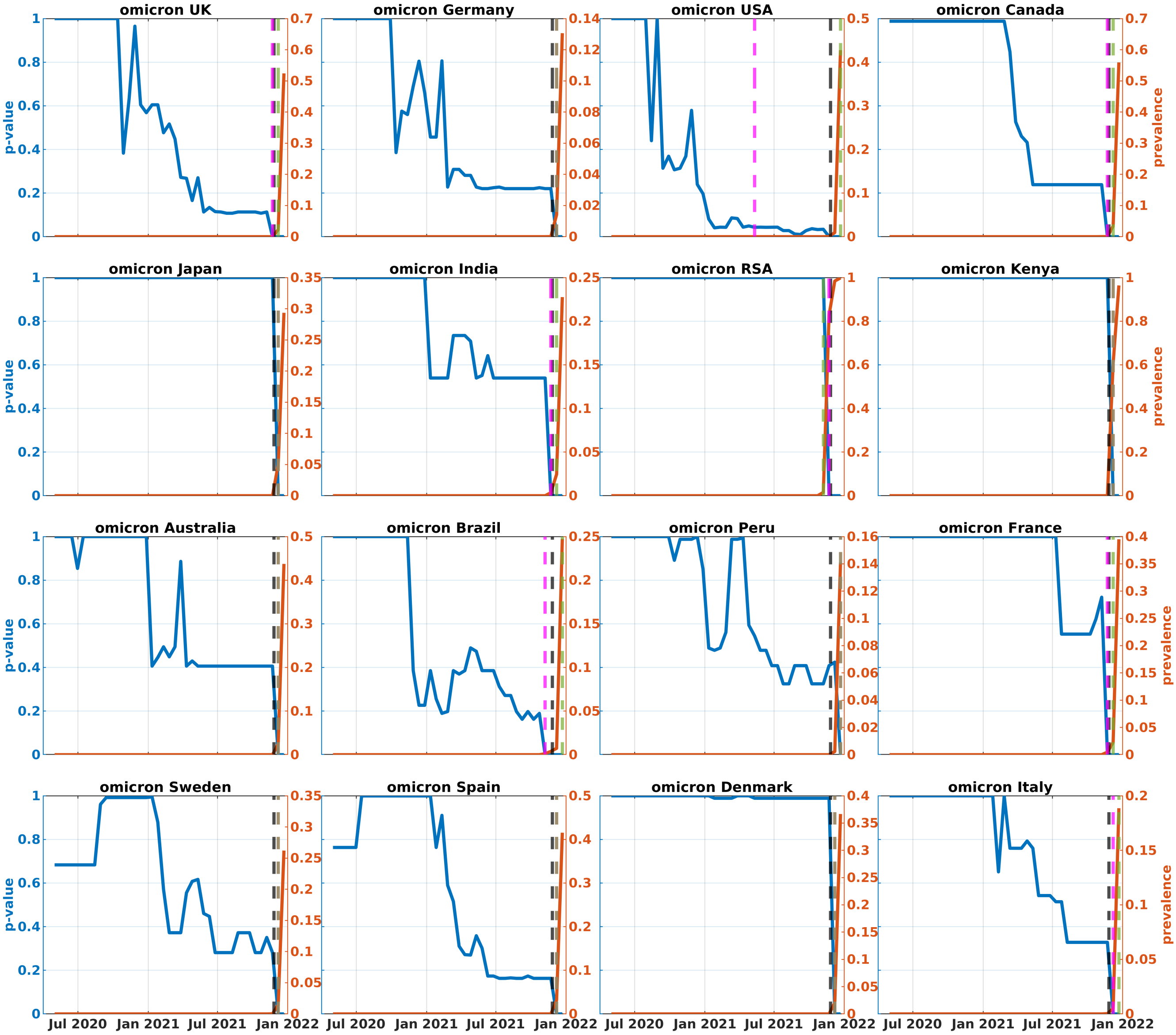

Supplementary Fig. 26: p-values (blue) and prevalences (red) of Omicron variant in the analyzed countries (second truncated dataset). Black, green, and magenta lines represent the times of VOC designation, achieving 1% prevalence, and becoming significantly dense, respectively.

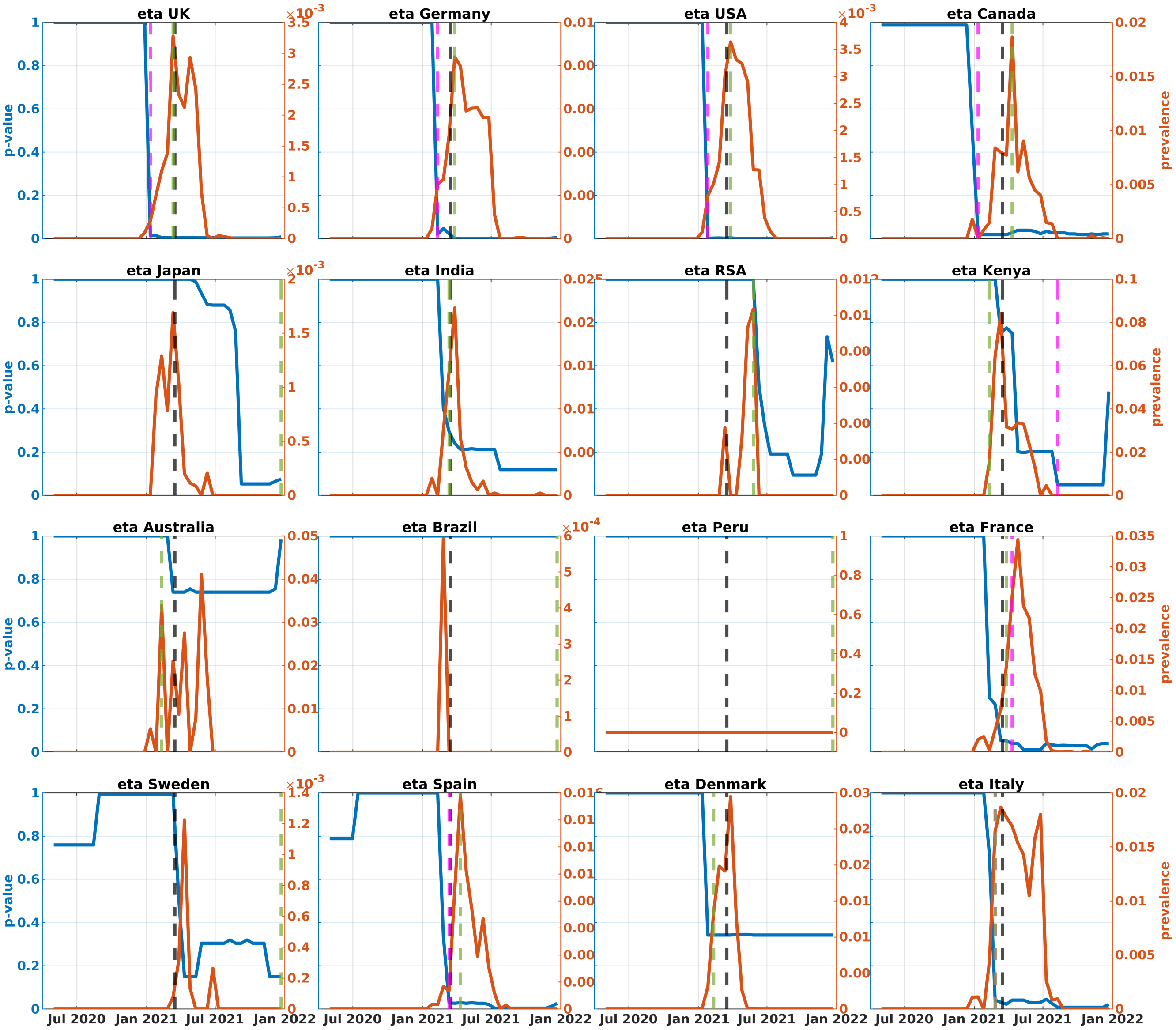

Supplementary Fig. 27: p-values (blue) and prevalences (red) of Eta variant in the analyzed countries (second truncated dataset). Black, green, and magenta lines represent the times of VOC designation, achieving 1% prevalence, and becoming significantly dense, respectively.

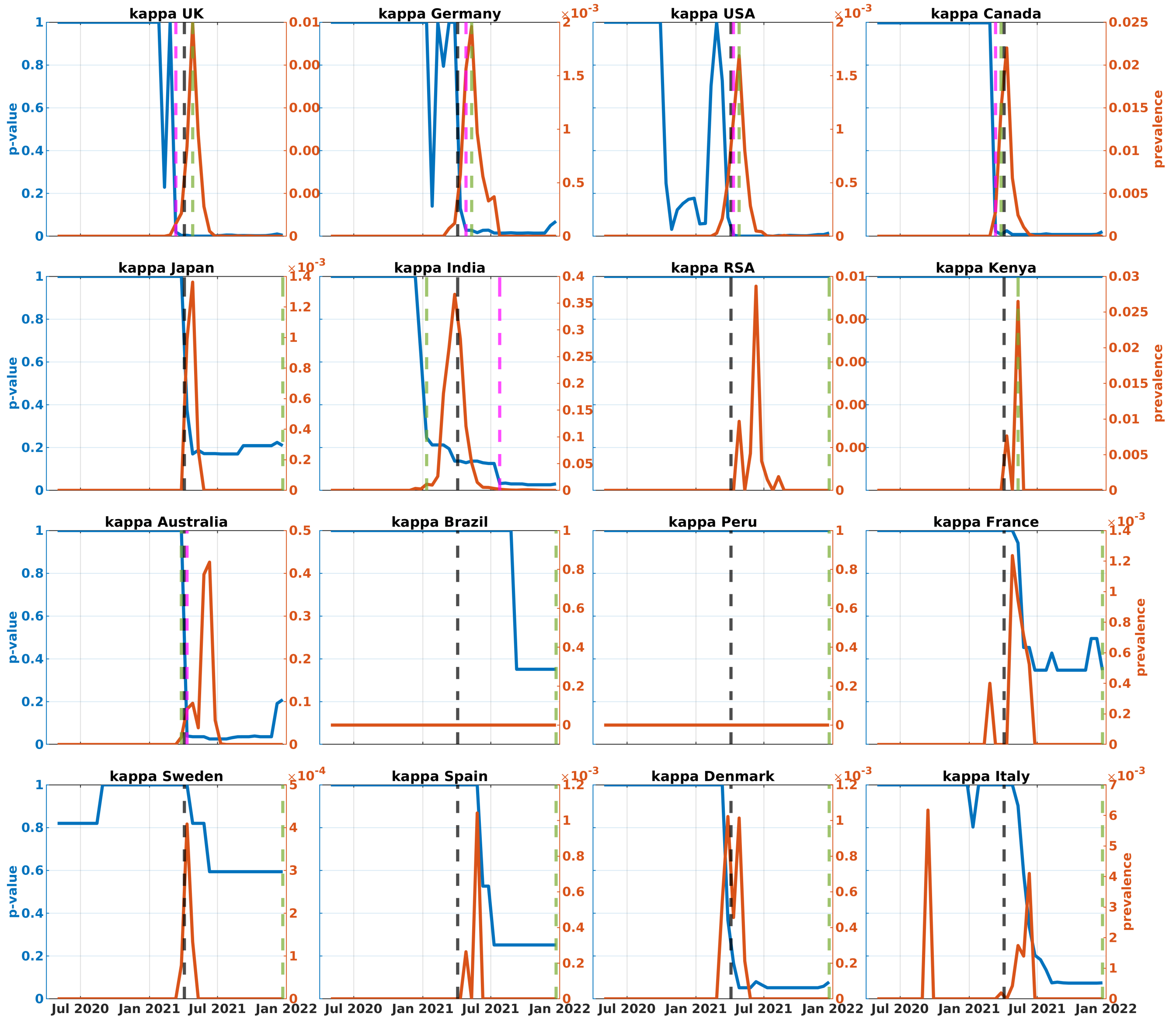

Supplementary Fig. 28: p-values (blue) and prevalences (red) of Kappa variant in the analyzed countries (second truncated dataset). Black, green, and magenta lines represent the times of VOC designation, achieving 1% prevalence, and becoming significantly dense, respectively.

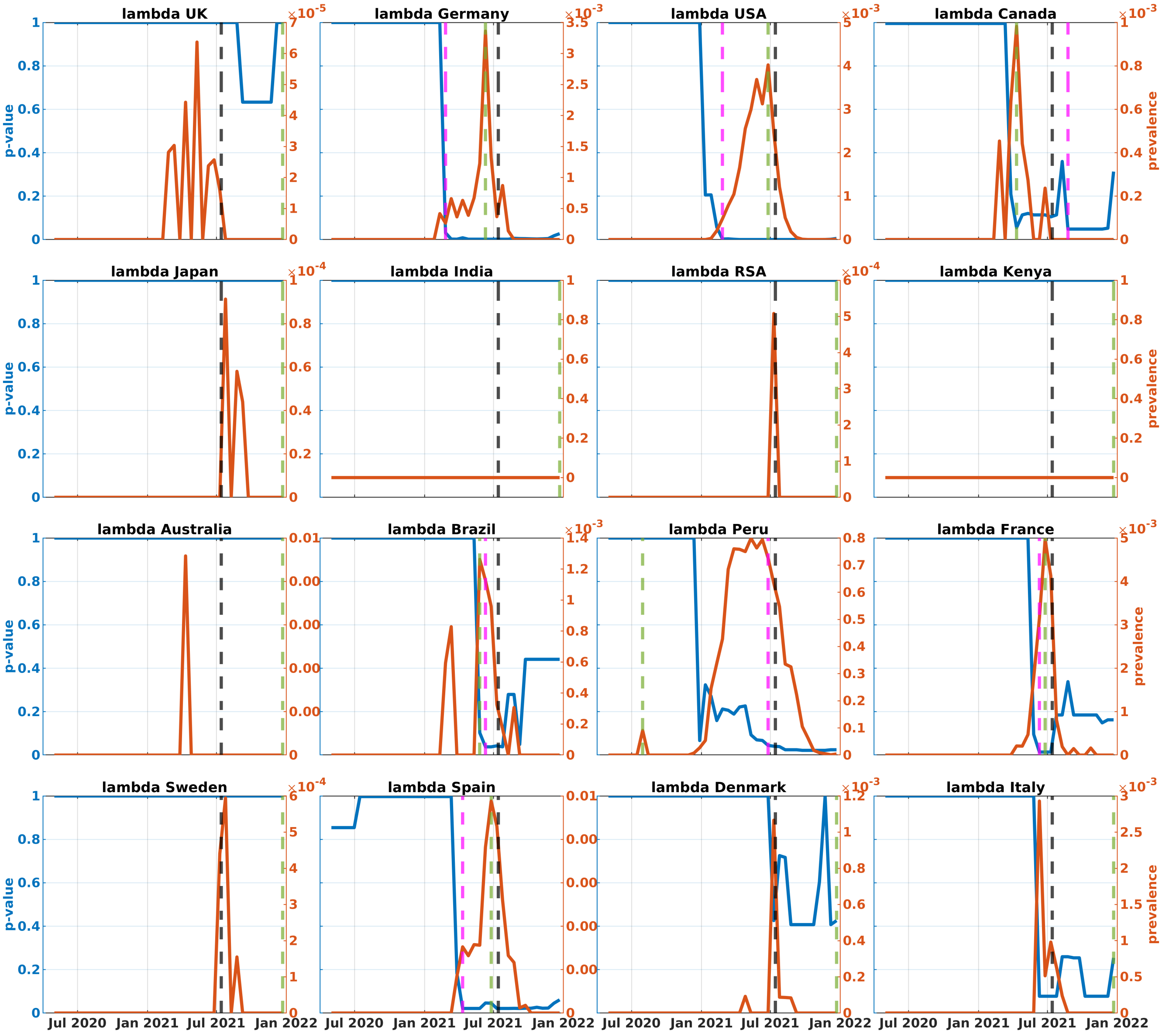

Supplementary Fig. 29: p-values (blue) and prevalences (red) of Lambda variant in the analyzed countries (second truncated dataset). Black, green, and magenta lines represent the times of VOC designation, achieving 1% prevalence, and becoming significantly dense, respectively.

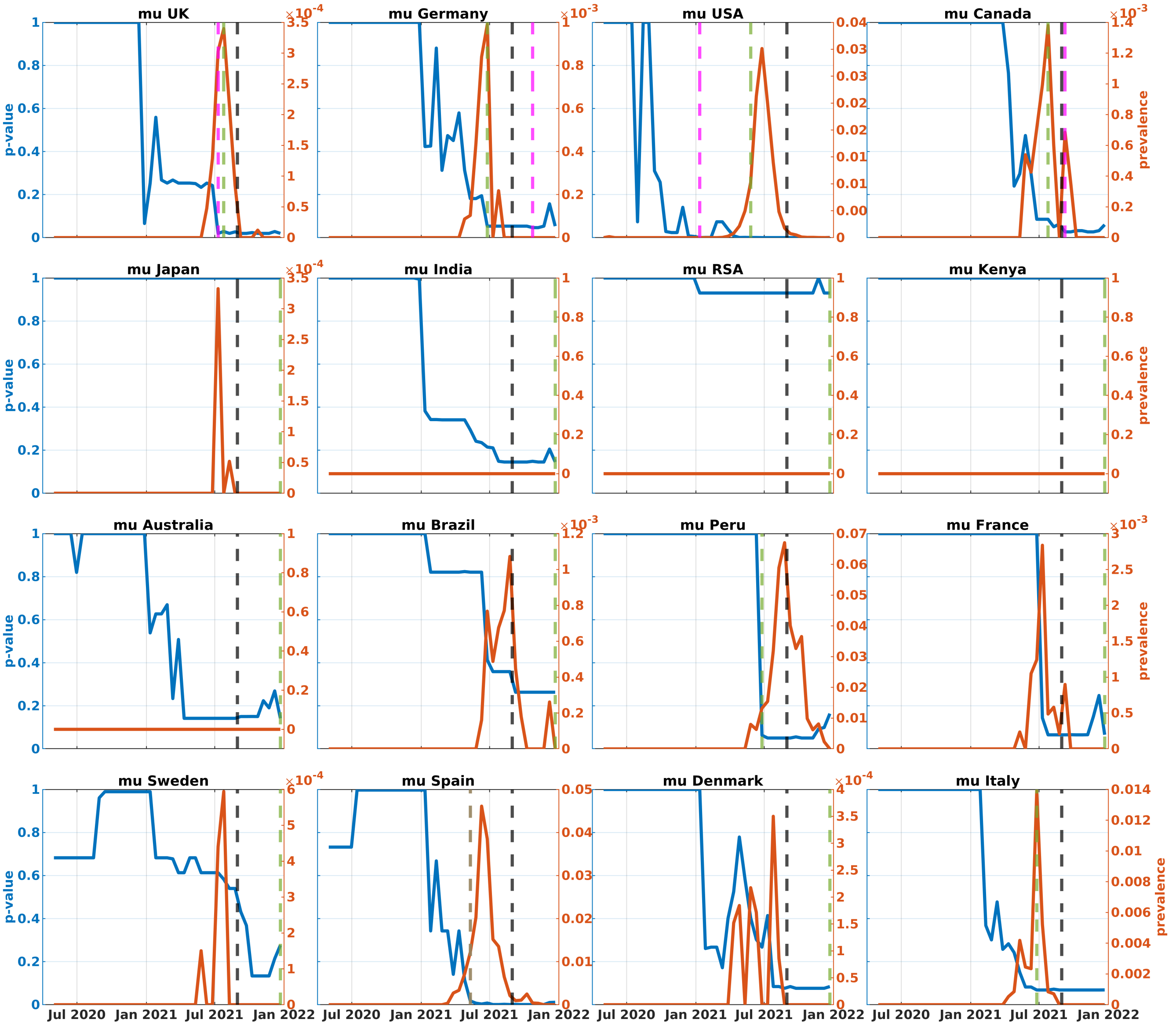

Supplementary Fig. 30: p-values (blue) and prevalences (red) of Mu variant in the analyzed countries (second truncated dataset). Black, green, and magenta lines represent the times of VOC designation, achieving 1% prevalence, and becoming significantly dense, respectively.

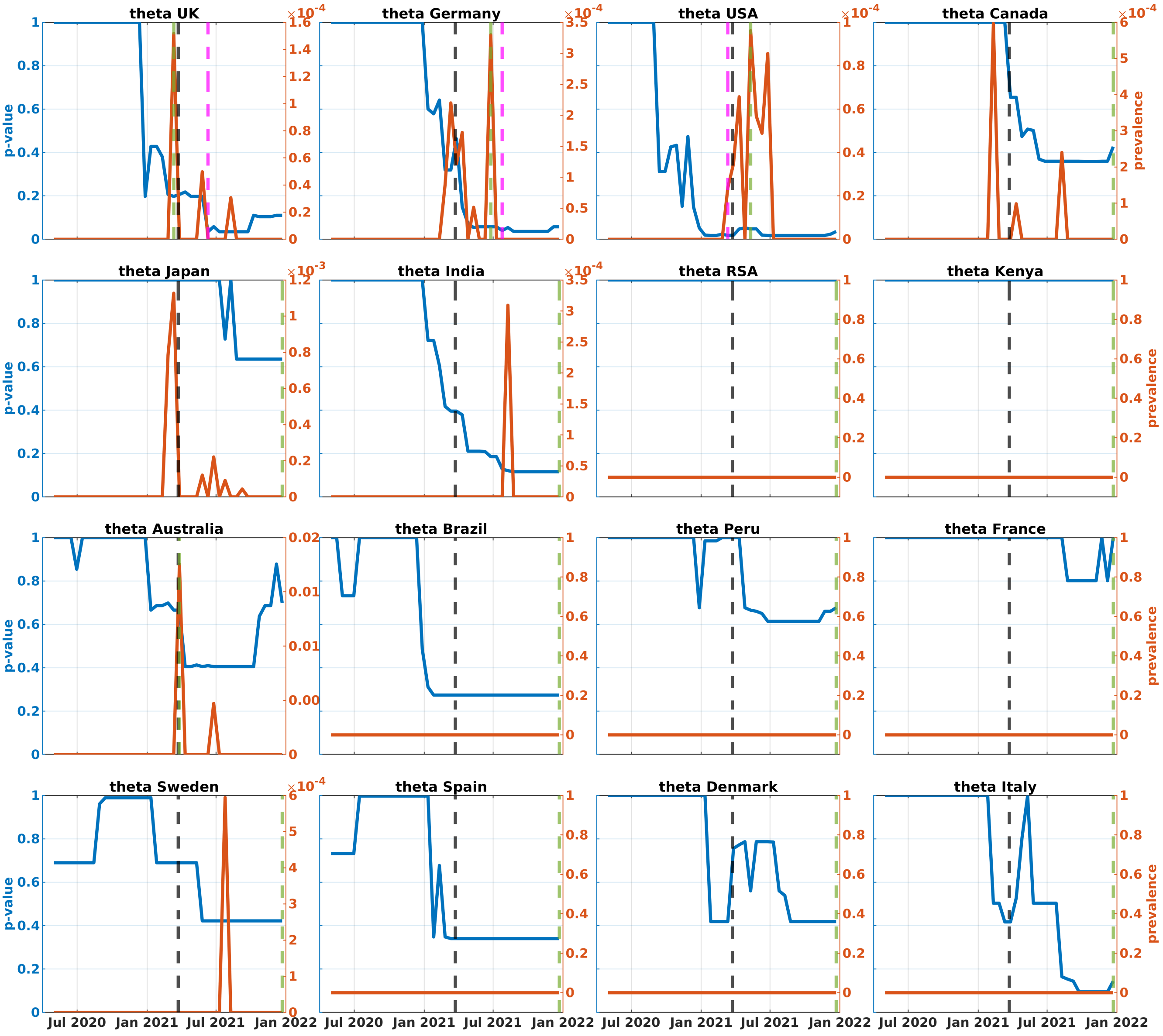

Supplementary Fig. 31: p-values (blue) and prevalences (red) of Theta variant in the analyzed countries (second truncated dataset). Black, green, and magenta lines represent the times of VOC designation, achieving 1% prevalence, and becoming significantly dense, respectively.

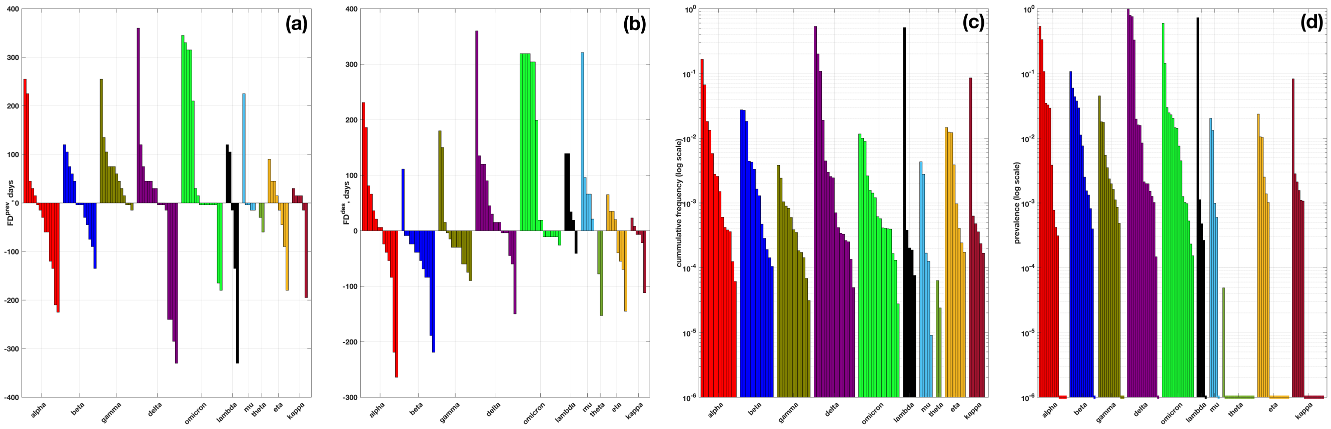

Supplementary Fig. 32: **Density-based adjusted** p**-values of VOCs/VOIs (complete dataset).** (b) and (c): Forecasting depths (y-axis) in relation to the 1% prevalence time and WHO designation time for each analyzed VOC/VOI across different countries. (d) and (e): Cumulative frequencies and prevalences for VOCs/VOIs across various countries at the times when they become significantly dense (in a logarithmic scale). Dashed lines at the bottom of the plot indicate that the variants reached significant density at frequencies/prevalences of 0.

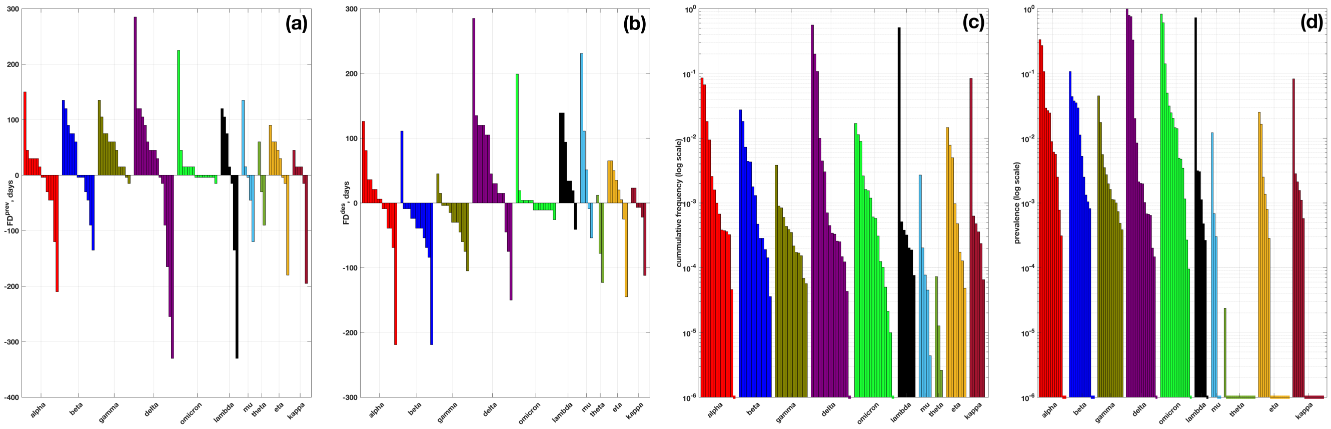

Supplementary Fig. 33: **Density-based adjusted** p**-values of VOCs/VOIs (second truncated dataset).** (b) and (c): Forecasting depths (y-axis) in relation to the 1% prevalence time and WHO designation time for each analyzed VOC/VOI across different countries. (d) and (e): Cumulative frequencies and prevalences for VOCs/VOIs across various countries at the times when they become significantly dense (in a logarithmic scale). Dashed lines at the bottom of the plot indicate that the variants reached significant density at frequencies/prevalences of 0.

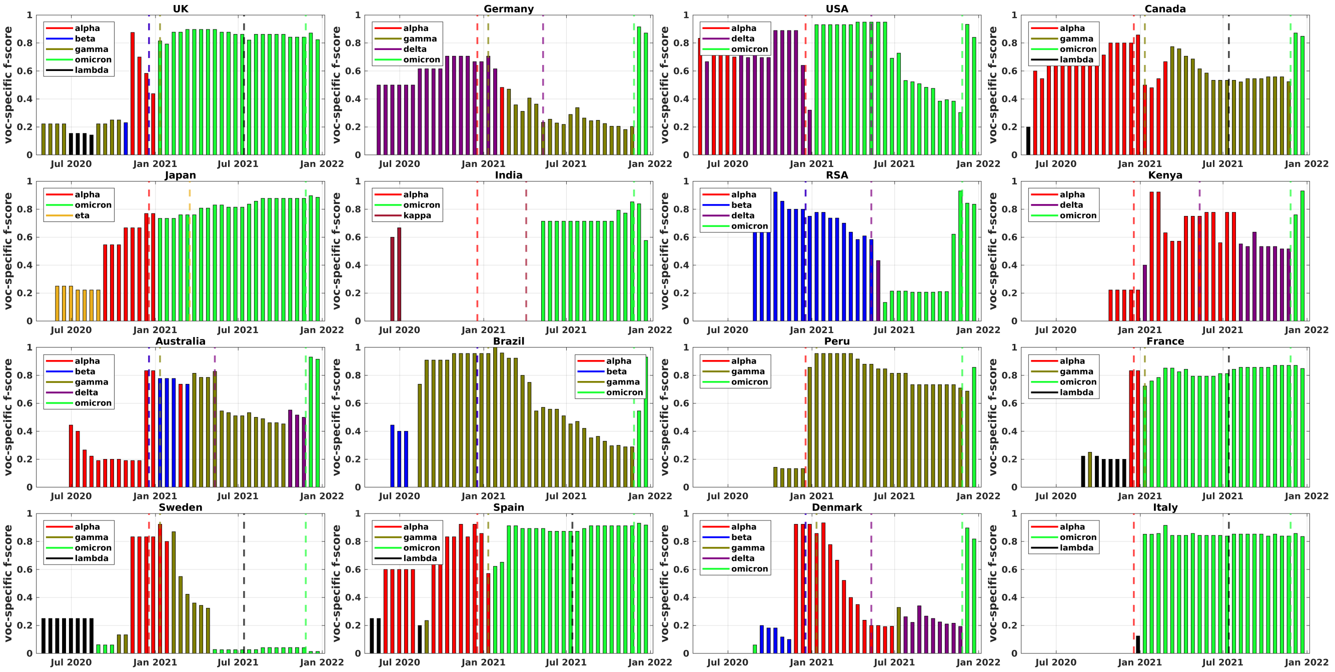

Supplementary Fig. 34: Comparison between VOCs and densest subnetworks of temporal epistatic networks for individual countries (complete dataset). At each time point, bar color code corresponds to the VOC closest to the inferred densest subnetwork, and the bar hight is equal to the respective f-score. Colored dashed lines mark times when specific VOCs were designated by WHO.

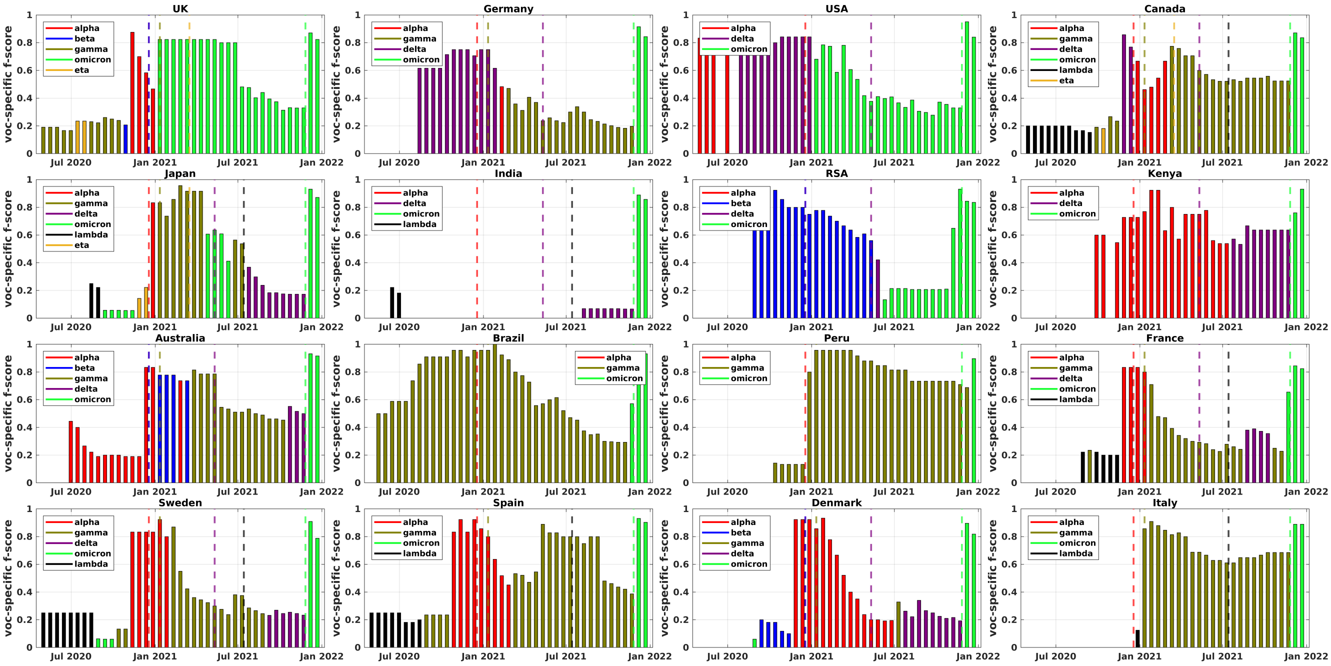

Supplementary Fig. 35: Comparison between VOCs and densest subnetworks of temporal epistatic networks for individual countries (first truncated dataset). At each time point, bar color code corresponds to the VOC closest to the inferred densest subnetwork, and the bar hight is equal to the respective f-score. Colored dashed lines mark times when specific VOCs were designated by WHO.

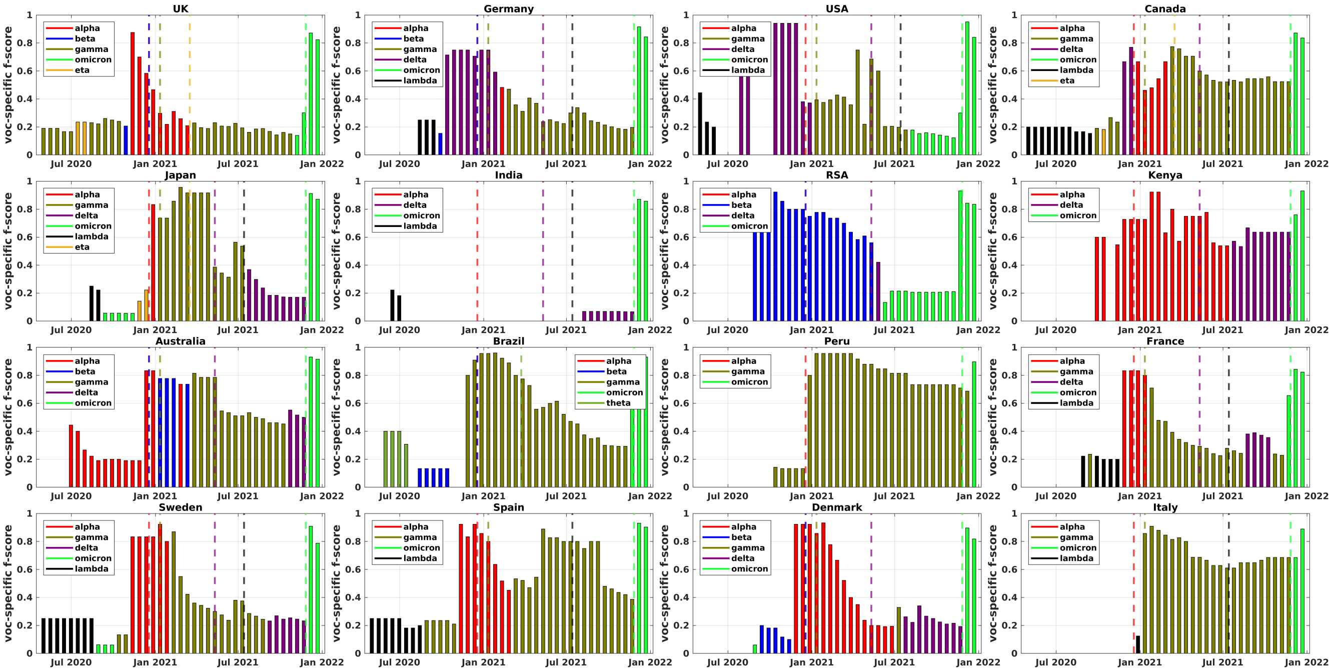

Supplementary Fig. 36: Comparison between VOCs and densest subnetworks of temporal epistatic networks for individual countries (second truncated dataset). At each time point, bar color code corresponds to the VOC closest to the inferred densest subnetwork, and the bar hight is equal to the respective f-score. Colored dashed lines mark times when specific VOCs were designated by WHO.

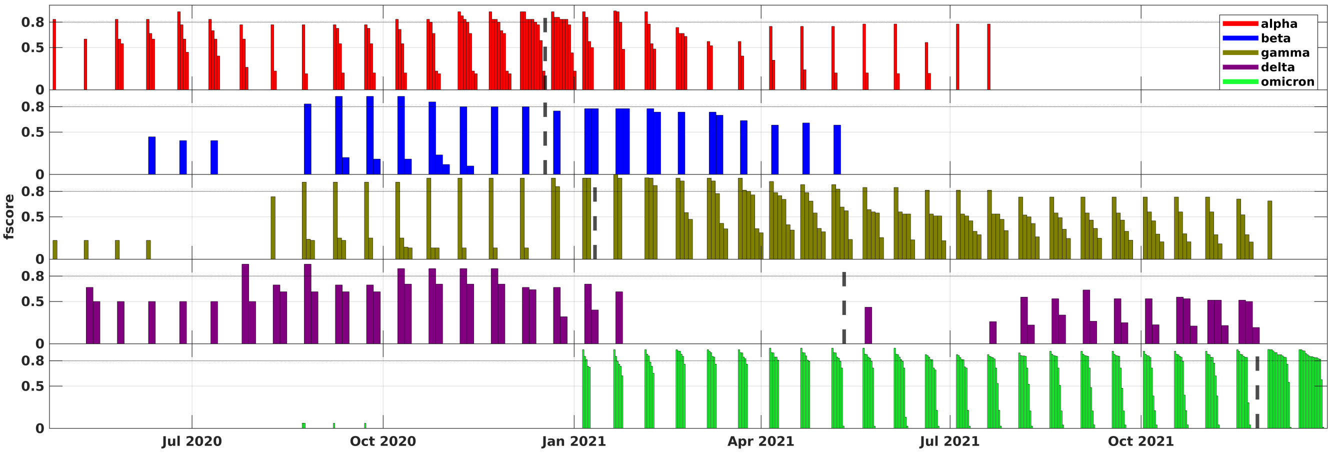

Supplementary Fig. 37: **Comparison of the densest subnetworks from temporal coordinated substitution networks (aggregated over 16 countries) with VOCs for the complete dataset.** Each bar in the plot represents a specific VOC. For every time point, the bars display the densest subgraphs from different countries that are most similar to that VOC, with the height of the bars indicating the corresponding f-scores. Colored dashed lines highlight the moments when the WHO designated the VOCs.

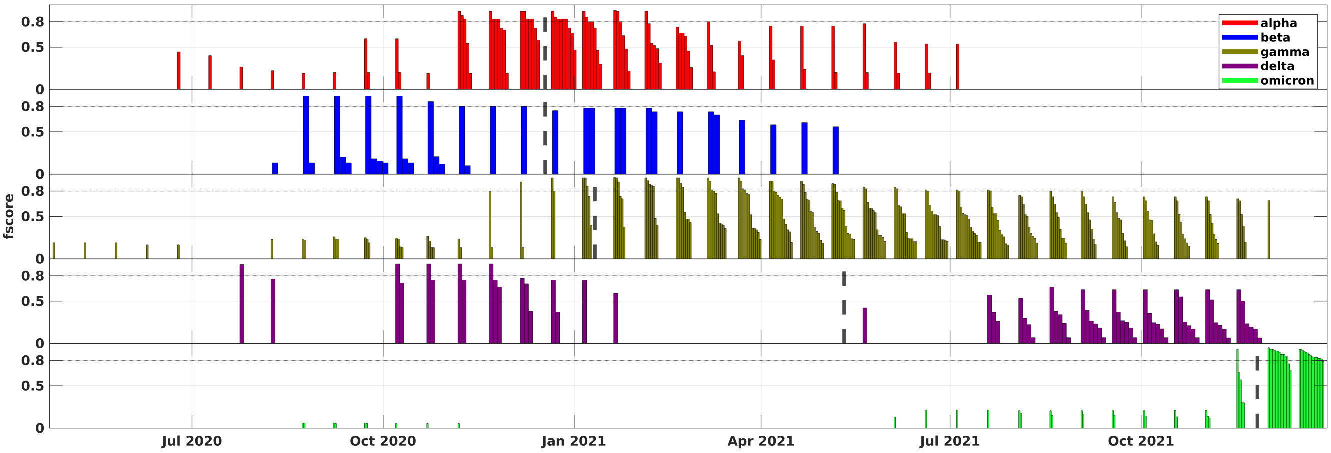

Supplementary Fig. 38: **Comparison of the densest subnetworks from temporal coordinated substitution networks (aggregated over 16 countries) with VOCs for the second truncated dataset.** Each bar in the plot represents a specific VOC. For every time point, the bars display the densest subgraphs from different countries that are most similar to that VOC, with the height of the bars indicating the corresponding f-scores. Colored dashed lines highlight the moments when the WHO designated the VOCs.

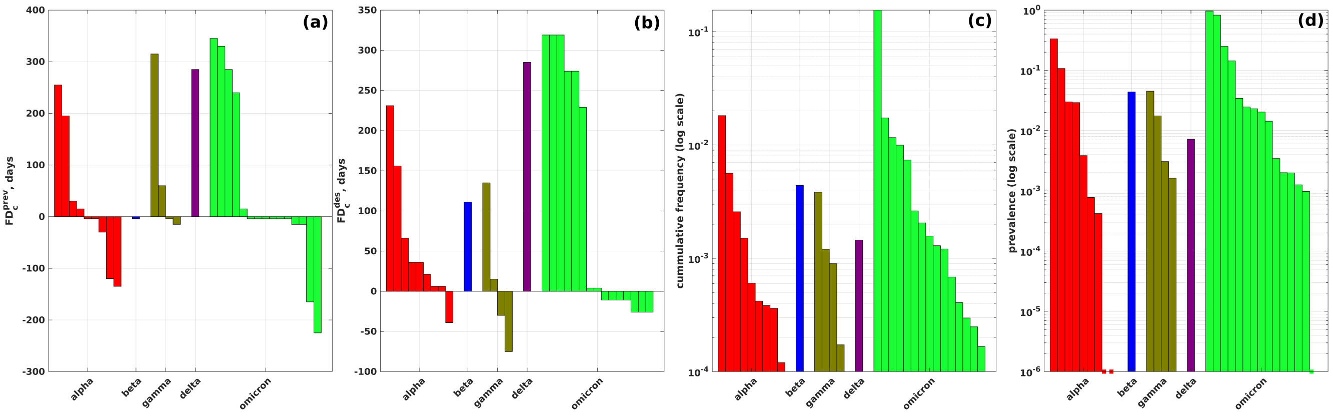

Supplementary Fig. 39: **Summary of comparison between VOCs and densest subnetworks of temporal epistatic networks for all countries (complete dataset)**. (a) and (b): forecasting depths (y-axis) with respect to the 1% prevalence time and WHO designation time for each analyzed VOCs over different countries. (c) and (d): cumulative frequencies and prevalences of VOCs over different countries at earliest times when they are at least 80% identical to densest subgraphs of epistatic networks (in logarithmic scale).

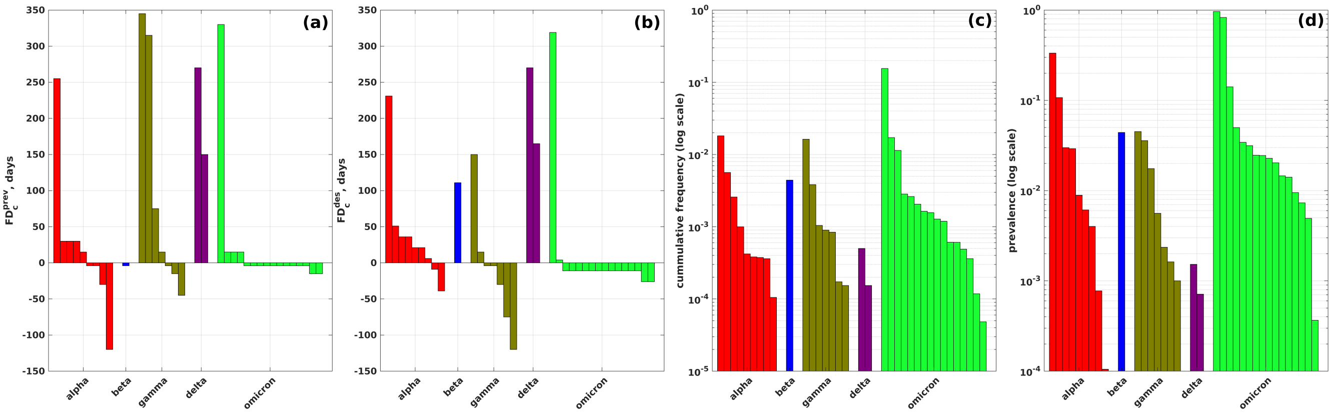

Supplementary Fig. 40: **Summary of comparison between VOCs and densest subnetworks of temporal epistatic networks for all countries (first truncated dataset)**. (a) and (b): forecasting depths (y-axis) with respect to the 1% prevalence time and WHO designation time for each analyzed VOCs over different countries. (c) and (d): cumulative frequencies and prevalences of VOCs over different countries at earliest times when they are at least 80% identical to densest subgraphs of epistatic networks (in logarithmic scale).

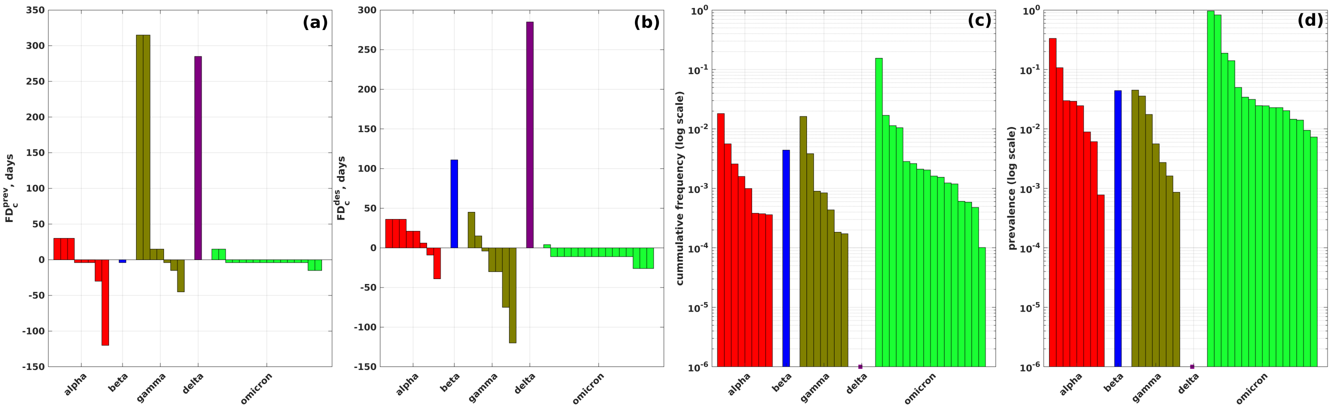

Supplementary Fig. 41: **Summary of comparison between VOCs and densest subnetworks of temporal epistatic networks for all countries (second truncated dataset)**. (a) and (b): forecasting depths (y-axis) with respect to the 1% prevalence time and WHO designation time for each analyzed VOCs over different countries. (c) and (d): cumulative frequencies and prevalences of VOCs over different countries at earliest times when they are at least 80% identical to densest subgraphs of epistatic networks (in logarithmic scale).

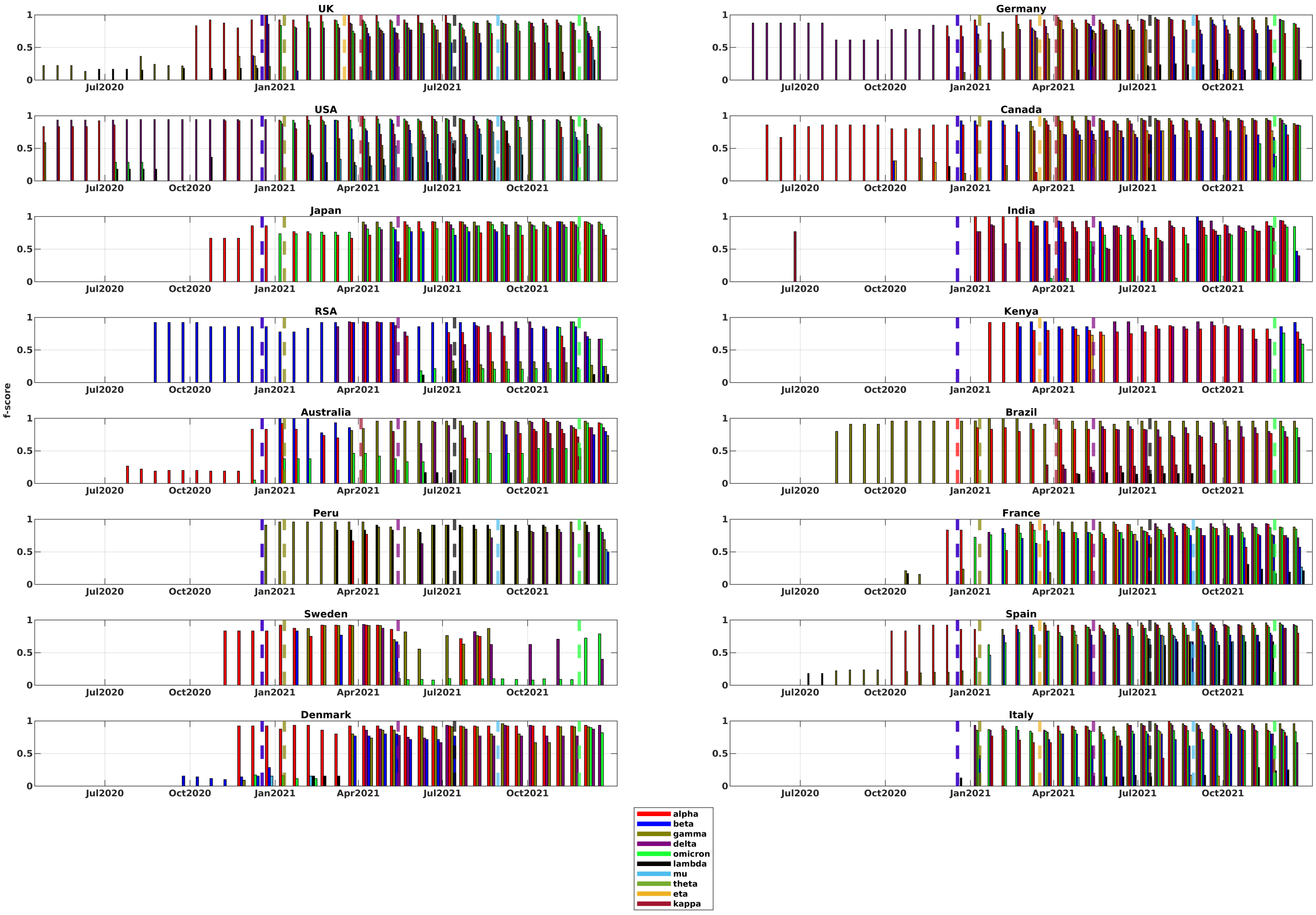

Supplementary Fig. 42: **Comparison between VOCs and inferred haplotypes for individual countries** (complete dataset). At each time point, each bar represents an inferred haplotype closest to a particular VOC, the bar height is equal to the respective f-score. Colored dashed lines mark times when specific VOCs/VOIs were designated by WHO.

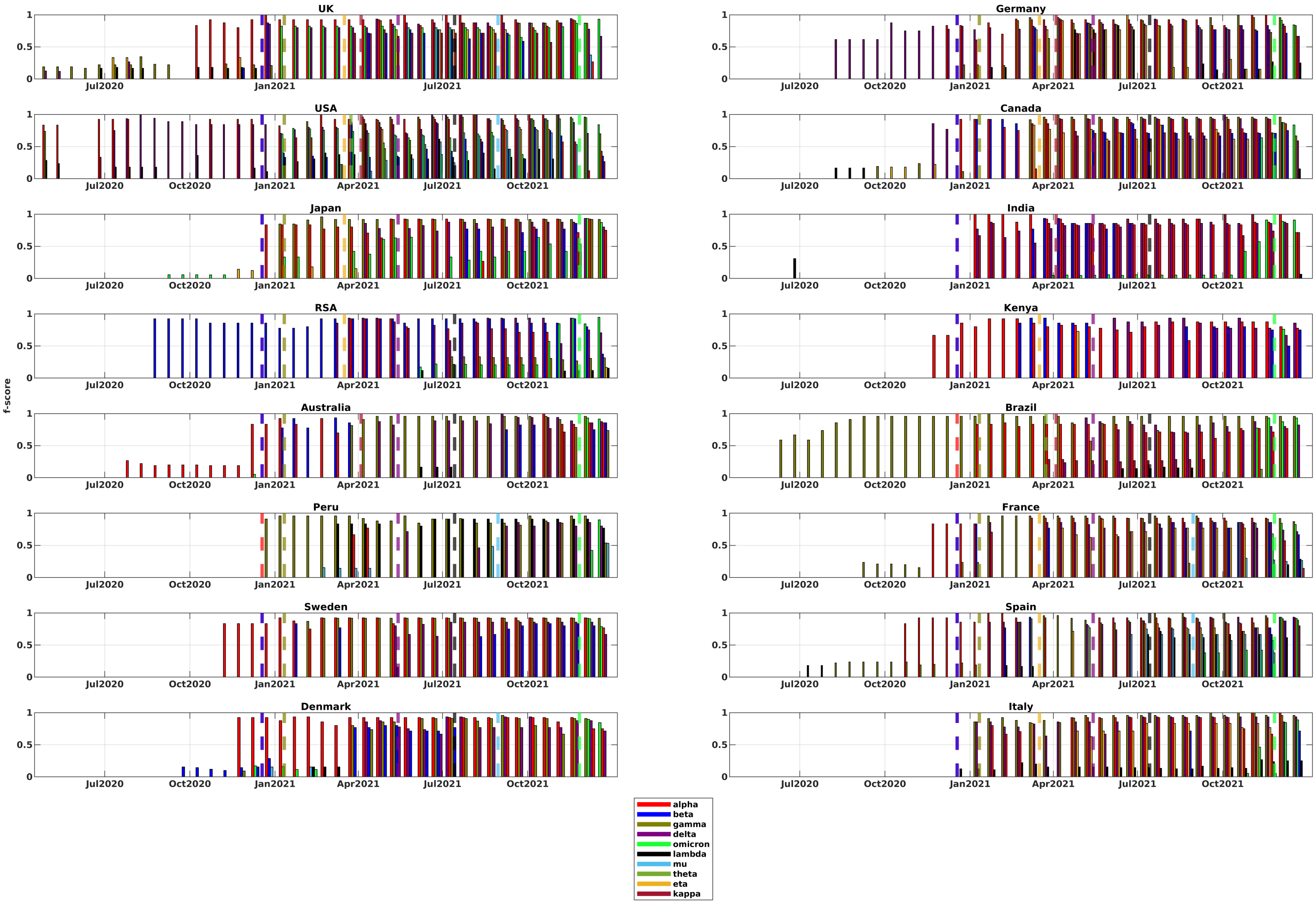

Supplementary Fig. 43: **Comparison between VOCs and inferred haplotypes for individual countries** (complete dataset). At each time point, each bar represents an inferred haplotype closest to a particular VOC, the bar height is equal to the respective f-score. Colored dashed lines mark times when specific VOCs/VOIs were designated by WHO.

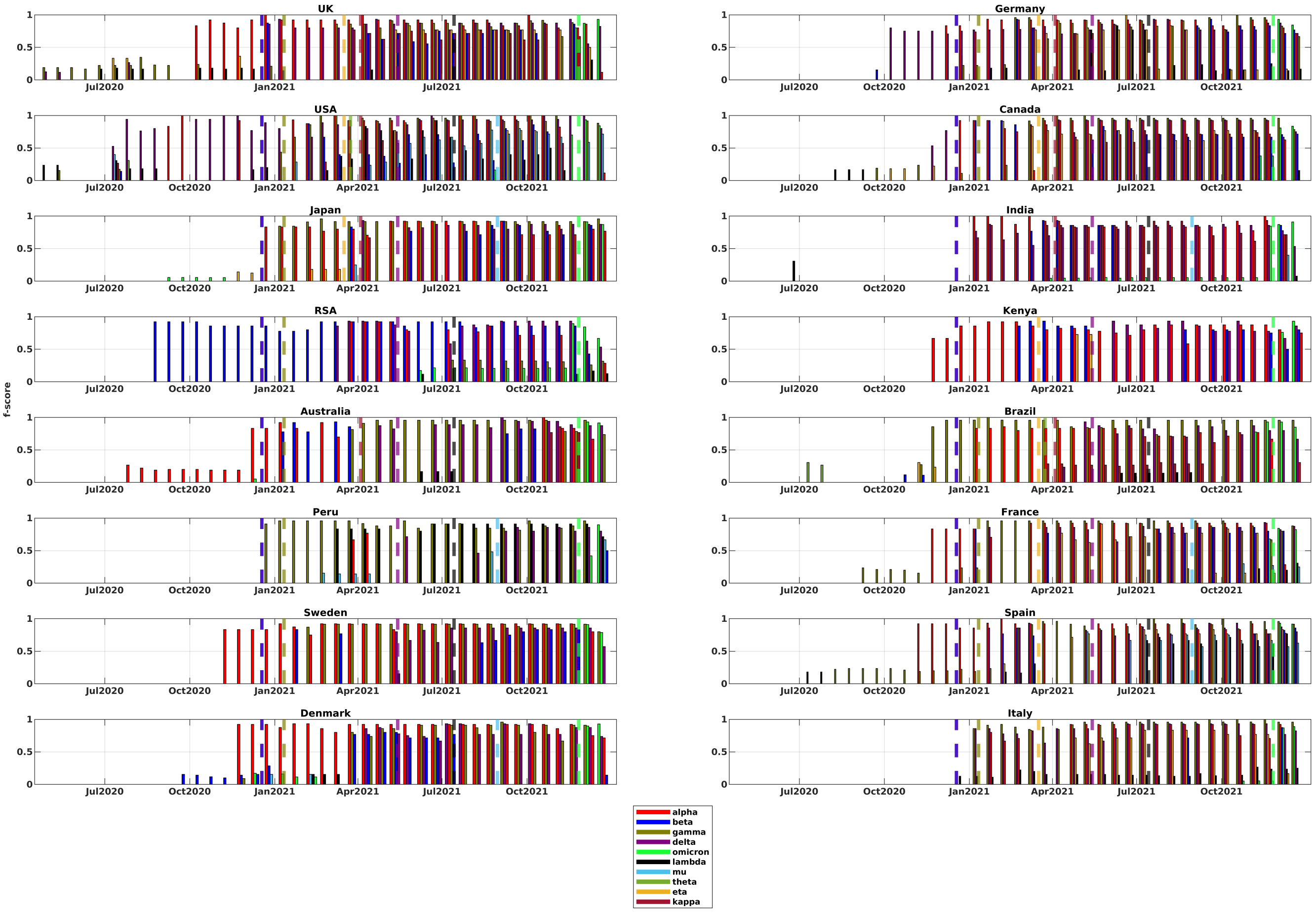

Supplementary Fig. 44: **Comparison between VOCs and inferred haplotypes for individual countries** (complete dataset). At each time point, each bar represents an inferred haplotype closest to a particular VOC, the bar height is equal to the respective f-score. Colored dashed lines mark times when specific VOCs/VOIs were designated by WHO.

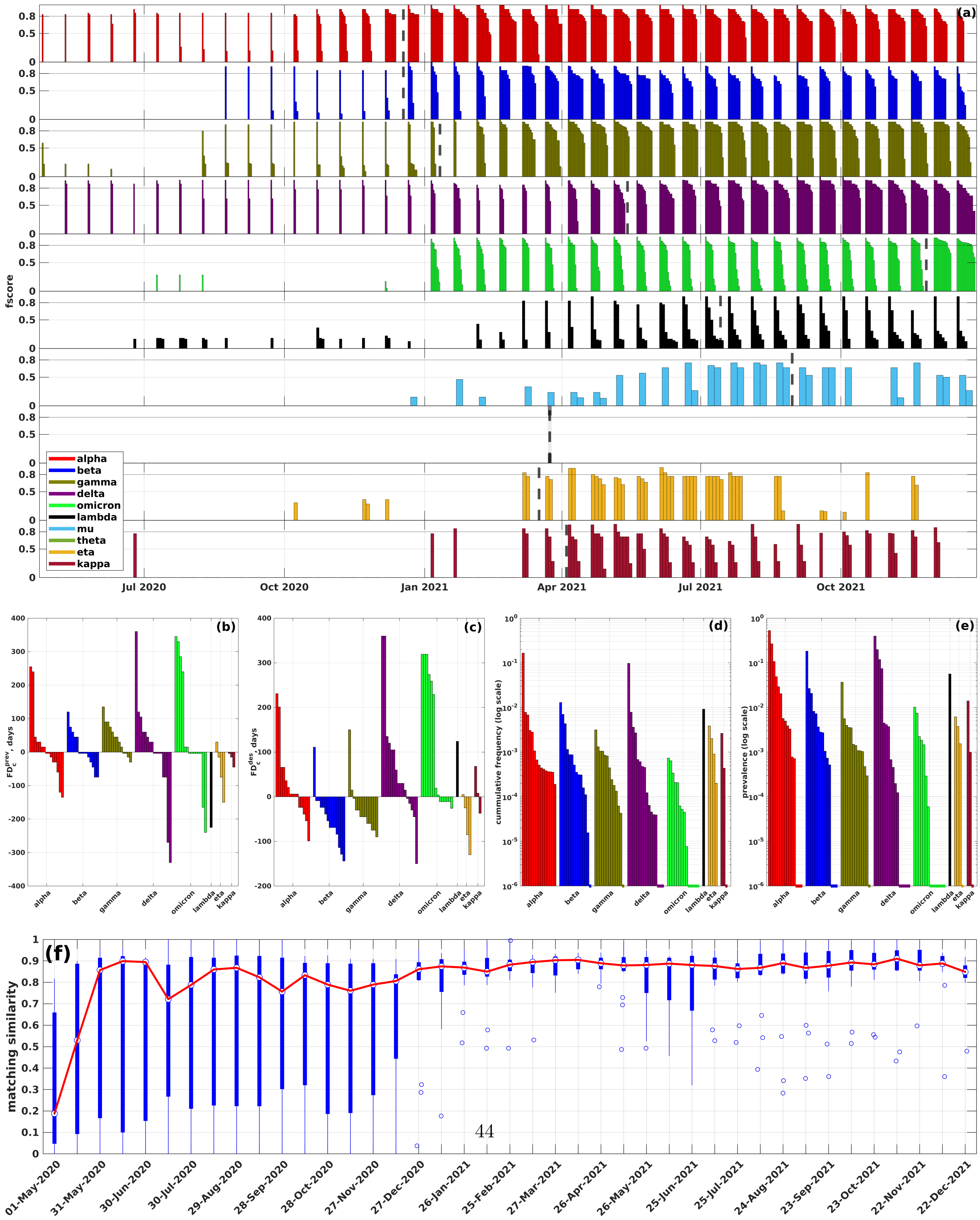

Supplementary Fig. 45: **(a) Summary of comparison between VOCs/VOIs and inferred haplotypes** (complete dataset). Each bar plot depicts the comparison results for a particular VOC/VOI; at each time point, bars correspond to inferred haplotypes from different countries closest to that VOC, and the bar heights are equal to the respective f-scores. Colored dashed lines mark times when the VOCs were designated by WHO. **(b) and (c): forecasting depths** (y-axis) with respect to the 1% prevalence time and WHO designation time for each analyzed VOCs/VOIs over different countries. **(d) and (e): cumulative frequencies and prevalences** of VOCs/VOIs over different countries at first variant call times (in logarithmic scale). Dashed lines at the bottom of the plot signify that the corresponding variants were detected at cumulative frequencies or prevalences 0. **(f) Precision of haplotype inference.** Blue box plot depicts summary statistics of matching similarity of $n = 16$ countries over $T = 21$ time points. The bottom and top of each box are the 25th and 75th percentiles, whiskers represent minimum and maximum values, white dot is a median. Red plot depicts the dynamics of median matching similarity over [illegible]

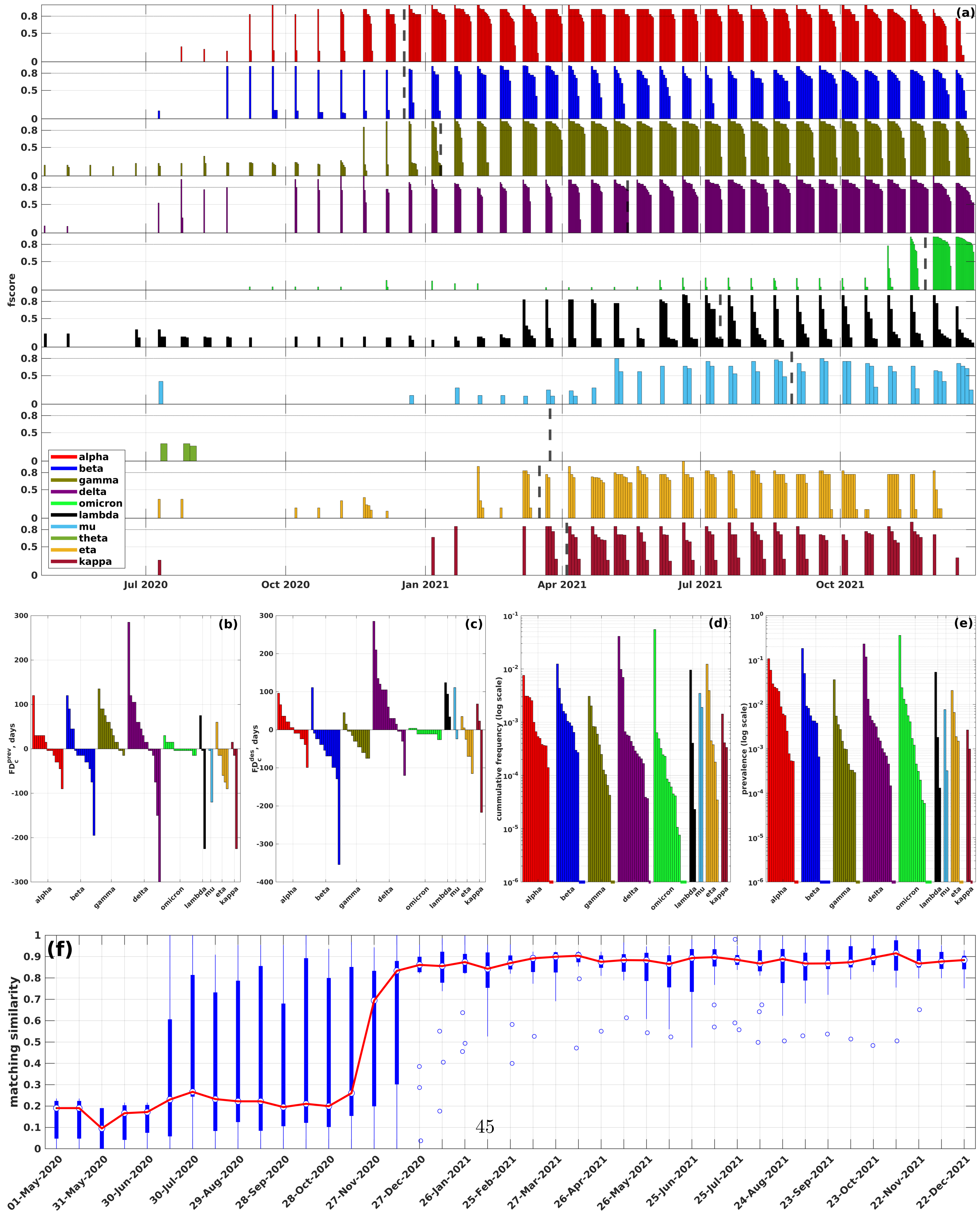

Supplementary Fig. 46: **(a) Summary of comparison between VOCs/VOIs and inferred haplotypes** (complete dataset). Each bar plot depicts the comparison results for a particular VOC/VOI; at each time point, bars correspond to inferred haplotypes from different countries closest to that VOC, and the bar heights are equal to the respective f-scores. Colored dashed lines mark times when the VOCs were designated by WHO. **(b) and (c): forecasting depths** (y-axis) with respect to the 1% prevalence time and WHO designation time for each analyzed VOCs/VOIs over different countries. **(d) and (e): cumulative frequencies and prevalences** of VOCs/VOIs over different countries at first variant call times (in logarithmic scale). Dashed lines at the bottom of the plot signify that the corresponding variants were detected at cumulative frequencies or prevalences 0. **(f) Precision of haplotype inference.** Blue box plot depicts summary statistics of matching similarity of $n = 16$ countries over $T = 21$ time points. The bottom and top of each box are the 25th and 75th percentiles, whiskers represent minimum and maximum values, white dot is a median. Red plot depicts the dynamics of median matching similarity over ti[illegible]

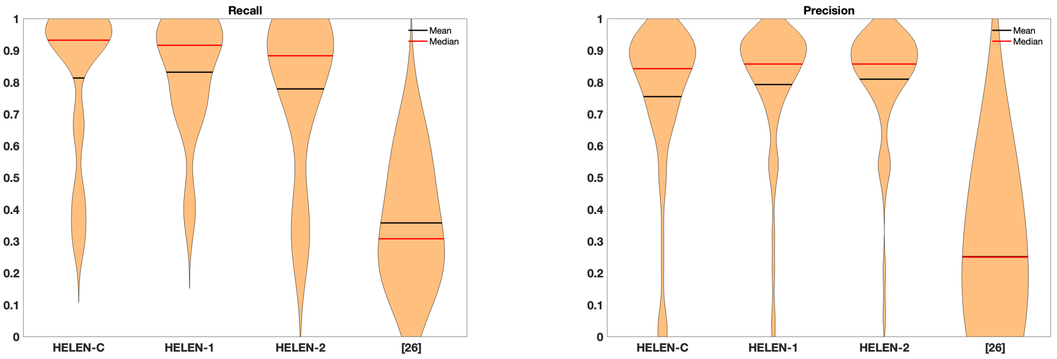

Supplementary Fig. 47: **Comparison of HELEN with the method from [26].** Distributions of recall values are visualized by violin plots, width of each violin at a given recall value represents the point density at that value. Median and mean values are highlighted by as red and black lines. HELEN-C, HELEN-1 and HELEN-2 denotes the results of HELEN for the complete, first and second truncated datasets. The study [26] reported the results for the complete dataset.
