# Supplementary material for: Early detection of emerging viral variants through analysis of community structure of coordinated substitution networks
Source: Nat Commun. 2024 Apr 2;15:2838. doi: 10.1038/s41467-024-47304-6 (PMC10987511; doi:10.1038/s41467-024-47304-6)
Supplement: Supplementary file 3 — Reporting Summary [file 41467_2024_47304_MOESM3_ESM.pdf]

nature portfolio

Corresponding author(s): Pavel Skums

Last updated by author(s): Feb 29, 2024

Reporting Summary

Nature Portfolio wishes to improve the reproducibility of the work that we publish. This form provides structure for consistency and transparency in reporting. For further information on Nature Portfolio policies, see our Editorial Policies and the Editorial Policy Checklist.

Statistics

For all statistical analyses, confirm that the following items are present in the figure legend, table legend, main text, or Methods section.

n/a	Confirmed	
☐	☒	The exact sample size (*n*) for each experimental group/condition, given as a discrete number and unit of measurement
☐	☒	A statement on whether measurements were taken from distinct samples or whether the same sample was measured repeatedly
☐	☒	The statistical test(s) used AND whether they are one- or two-sided<br>*Only common tests should be described solely by name; describe more complex techniques in the Methods section.*
☒	☐	A description of all covariates tested
☐	☒	A description of any assumptions or corrections, such as tests of normality and adjustment for multiple comparisons
☐	☒	A full description of the statistical parameters including central tendency (e.g. means) or other basic estimates (e.g. regression coefficient) AND variation (e.g. standard deviation) or associated estimates of uncertainty (e.g. confidence intervals)
☐	☒	For null hypothesis testing, the test statistic (e.g. *F*, *t*, *r*) with confidence intervals, effect sizes, degrees of freedom and *P* value noted<br>*Give P values as exact values whenever suitable.*
☒	☐	For Bayesian analysis, information on the choice of priors and Markov chain Monte Carlo settings
☒	☐	For hierarchical and complex designs, identification of the appropriate level for tests and full reporting of outcomes
☒	☐	Estimates of effect sizes (e.g. Cohen's *d*, Pearson's *r*), indicating how they were calculated

Our web collection on statistics for biologists contains articles on many of the points above.

Software and code

Policy information about availability of computer code

Data collection	No software was used for data collection.
Data analysis	Data analysis was done using Matlab R2023a and Gurobi 10.0.3. Custom code is available at https://github.com/compbel/HELEN

For manuscripts utilizing custom algorithms or software that are central to the research but not yet described in published literature, software must be made available to editors and reviewers. We strongly encourage code deposition in a community repository (e.g. GitHub). See the Nature Portfolio guidelines for submitting code & software for further information.

Data

Policy information about availability of data

All manuscripts must include a data availability statement. This statement should provide the following information, where applicable:

- Accession codes, unique identifiers, or web links for publicly available datasets
- A description of any restrictions on data availability
- For clinical datasets or third party data, please ensure that the statement adheres to our policy

The findings of this study are based on genomic data available on GISAID (https://gisaid.org/); sequence accession numbers can be found at https://epicov.org/epi3/epi_set/230407vq?main=true. The generated secondary data is available at https://github.com/compbel/HELEN (DOI: 10.5281/zenodo.10695159).

Research involving human participants, their data, or biological material

Policy information about studies with human participants or human data. See also policy information about sex, gender (identity/presentation), and sexual orientation and race, ethnicity and racism.

Reporting on sex and gender	N/A
Reporting on race, ethnicity, or other socially relevant groupings	N/A
Population characteristics	N/A
Recruitment	N/A
Ethics oversight	N/A

Note that full information on the approval of the study protocol must also be provided in the manuscript.

Field-specific reporting

Please select the one below that is the best fit for your research. If you are not sure, read the appropriate sections before making your selection.

☐ Life sciences ☐ Behavioural & social sciences ☒ Ecological, evolutionary & environmental sciences

For a reference copy of the document with all sections, see nature.com/documents/nr-reporting-summary-flat.pdf

Ecological, evolutionary & environmental sciences study design

All studies must disclose on these points even when the disclosure is negative.

Study description	The study demonstrates that by examining the community structure of viral coordinated substitution networks, it is feasible to efficiently detect or predict emerging haplotypes with altered transmissibility. These haplotypes can be linked to dense network communities, which become discernible earlier than their associated viral variants reach noticeable prevalence levels. From these insights, we developed HELEN (Heralding Emerging Lineages in Epistatic Networks), a computational framework that identifies densely connected communities of SAV alleles and merges them into haplotypes using a combination of statistical inference, population genetics, and discrete optimization techniques.
Research sample	SARS-CoV-2 genomic data were downloaded from GISAID (https://gisaid.org/). Since the detection of linked pairs of SAVs and dense communities in coordinated substitution networks is affected by the number of sequences, we selected for analysis data from 16 countries with the largest sample sizes, while maintaining geographic diversity. Thus, we selected two countries per continent (excluding Oceania where 1 country was selected) with the largest numbers of spike amino acid sequences sampled over the considered time period, complemented by 5 extra countries with the largest samples.
Sampling strategy	All publicly available genomic data from selected countries within the study time frame were analyzed.
Data collection	The data analyzed in this study were downloaded by the authors from the GISAID repository (https://gisaid.org/) through its web interface. Upon download, the dataset was organized by country and collection date using Matlab version R2023a.
Timing and spatial scale	This study analyzes the data with GISAID-reported collection dates between January 4, 2020 and December 31, 2021. Data was uploaded to GISAID daily, and thus the day was used as the basic time unit. For the clarity of reporting and visualization, the results in figures and tables are reported at uniformly distributed time points with 14-day difference between consecutive points. Sampling frequency and periodicity are not pertinent as we analyzed the entirety of available data without subsampling. The analysis and reporting of results were conducted at the country level.
Data exclusions	All available data were analyzed
Reproducibility	Software tools developed within this study are freely available at https://github.com/compbel/HELEN and can be independently run to validate the study findings.
Randomization	Randomization is not applicable to this study, since we were not evaluating differences between groups . This study relied on analyses of all publicly available data from GISAID that was collected by data submitters during the analyzed time period in the 16 analyzed countries.
Blinding	Blinding is not applicable to this study. It does not involve a clinical trial design and does not compare treatment effects with a control group.

Did the study involve field work? ☐ Yes ☒ No

Reporting for specific materials, systems and methods

We require information from authors about some types of materials, experimental systems and methods used in many studies. Here, indicate whether each material, system or method listed is relevant to your study. If you are not sure if a list item applies to your research, read the appropriate section before selecting a response.

Materials & experimental systems

n/a	Involved in the study
☒	☐ Antibodies
☒	☐ Eukaryotic cell lines
☒	☐ Palaeontology and archaeology
☒	☐ Animals and other organisms
☒	☐ Clinical data
☒	☐ Dual use research of concern
☒	☐ Plants

Methods

n/a	Involved in the study
☒	☐ ChIP-seq
☒	☐ Flow cytometry
☒	☐ MRI-based neuroimaging

Plants

Seed stocks	N/A
Novel plant genotypes	N/A
Authentication	N/A
